# Testing for methicillin-resistant *Staphylococcus aureus* in the anterior nares for antibiotic de-escalation in patients presenting with acute skin and soft tissue infections: systematic review and meta-analysis

**DOI:** 10.1017/ice.2025.58

**Published:** 2025-07

**Authors:** Sara Bohjanen, Connor Goldstick, Maria Hordinsky

**Affiliations:** 1 Department of Dermatology, University of Minnesota, Minneapolis, MN, USA; 2 Department of Economics, University of California San Diego, San Diego, CA, USA

## Abstract

**Objective:**

To improve the understanding of appropriate antibiotic de-escalation and stewardship by consolidating the evidence on screening for methicillin-resistant *Staphylococcus aureus* (MRSA) colonization of the nares to predict MRSA in acute skin and soft tissue infections (SSTIs).

**Methods:**

This meta-analysis was performed according to PRISMA guidelines. Six databases were searched by two reviewers for articles on MRSA detection in the nares and acute SSTIs. The quality and risk of bias of the articles were then assessed. The primary outcomes of interest were pooled sensitivity and specificity. Sub-analyses were also performed to test for heterogeneity.

**Results:**

After screening 1040 records, 15 articles (n = 1,970) were included in the meta-analysis. Using MRSA nares screening to predict MRSA in acute SSTIs had an overall specificity of 0.949 and sensitivity of 0.474. With a prevalence of 29.1%, the calculated NPV was 0.815. There were sub-analyses on various study variables, such as study location, participant age, and detection by polymerase chain reaction versus culture. The only significant finding was an increased sensitivity for adults (0.543) compared to pediatric participants (0.285).

**Discussion:**

To our knowledge, this is the first meta-analysis that focuses on the performance of MRSA nares screening for predicting MRSA infection in patients presenting with acute SSTIs. The baseline prevalence of MRSA SSTIs is important for interpreting the screening results, and the prevalence is influenced by geography and patient factors. This clinical context must be considered before utilizing MRSA nares screening for acute SSTIs.

## Introduction

Skin and soft tissue infections (SSTIs) are common worldwide^1^ and can cause significant morbidity and mortality.^2^ *Staphylococcus aureus* is the causative organism in a large portion of SSTIs, and this includes antibiotic-resistant strains like methicillin-resistant *Staphylococcus aureus* (MRSA).^3^ MRSA-targeting antibiotics have a risk of major adverse reactions, including clindamycin-associated *Clostridioides difficile* infection, vancomycin flushing syndrome, and renal toxicity.^4^ Antibiotic de-escalation after empiric treatment with MRSA-targeting agents is often desired, usually occurring after susceptibilities return. Nasal swab polymerase chain reaction (PCR) detection of MRSA has a relatively quick turn-around time, and this screening and identification of MRSA-colonized patients has been proposed as a tool for antibiotic de-escalation.^5–8^

Screening for MRSA in the anterior nares has a high specificity for diabetic foot infections^5^ and MRSA pneumonia.^7^ However, the relationship between MRSA colonization of the nose and whether MRSA is the causative organism for patients presenting with SSTIs is less well-characterized. Previous studies reviewed the literature on the predictive value of MRSA colonization for many types of infections, including SSTIs, but they did not report nares-only data, and they included infections that developed during hospitalization.^6,8^ Healthcare-associated MRSA and community-associated MRSA have different genotypes, virulence factors, resistance profiles, and clinical characteristics.^9^ By only including patients who present with acute SSTIs, we can select for predominately (although not exclusively) community-associated infections. To our knowledge, our article describes the first meta-analysis that focuses on the utility of MRSA detection in the anterior nares for predicting MRSA infection among patients presenting with an acute SSTI.

## Methods

This review was conducted according to PRISMA guidelines,^10^ and the protocol was prospectively registered with PROSPERO (CRD42024524640). Six databases (PubMed, MEDLINE via Ovid, EMBASE via Ovid, Web of Science, Scopus, and the Cochrane Library) were searched from date of first entry through January 1, 2025. Title, abstract, and keywords were searched for terms including methicillin-resistant *Staphylococcus aureus* (or MRSA), skin (or cutaneous or derm*), skin and soft tissue infection (or SSTI or pyoderma or skin infection), and nasal (or nose or nares). The exact searches are described in Supplementary Table 1. The search was restricted to English language articles with human participants, and there were no publication date restrictions. Published and peer-reviewed studies, including observational or randomized controlled trials, were included. To avoid undefined estimates of the screening test, case reports and case series were excluded. Review articles,, conference abstracts, and grey literature were also excluded.

Studies were eligible for inclusion if: (1) they included data on the MRSA nasal carriage status of at least 15 people presenting with acute SSTIs; (2) samples of the nares and the SSTIs were obtained within 72 hours of each other; and (3) the reports included enough information to form a 2×2 contingency table. To ensure the meta-analysis results were representative of a random patient presenting with an acute SSTI and, thus more generalizable, we excluded studies if the inclusion criteria required participants to have a particular comorbid disease/illness, a specific previous treatment, or a history of MRSA infection/colonization (or lack thereof).

After de-duplicating search results, two reviewers (SB, CG) screened the titles and abstracts for relevance and for meeting the above criteria. We screened the full text of each remaining article according to the same criteria. We hand-searched the reference lists of retained articles for additional articles. For included studies, we utilized a standardized form for data collection on the methods, participant demographics, and the 2×2 table (see supplementary material). Each selected study also had a risk of bias assessment using the revised Quality Assessment of Diagnostic Accuracy Studies tool (QUADAS-2)^11^ (see supplementary material). If there was a high risk of bias in at least two of the four domains, then the studies were excluded. Throughout this process, we resolved disagreements between reviewers by consensus.

The main outcome of interest was the pooled sensitivity and specificity of the nares MRSA status for predicting SSTI MRSA status. A hierarchical bivariate model without covariates, which is numerically equivalent to the hierarchical SROC model without covariates,^12^ estimated the pooled sensitivity and specificity. The model takes into account how different studies may have calibrated their tests differently to obtain disparate sensitivity and specificity results. We used MetaDTA, an online Rshiny app,^13,14^ to estimate and visualize the model. To examine heterogeneity, we completed sub-group analyses on subgroups of participants (pediatric vs adult patients) or methods (PCR vs culture) that included at least 100 people. We used a hierarchical bivariate model to estimate the heterogeneous sensitivity and specificity. We used the R package rjags^15^ to estimate and visualize the heterogeneous models.

## Results

We screened the titles and abstracts of 1040 publications, with 205 remaining for full text review (see Supplementary Figure 1 for flow diagram). We excluded records based on the criteria described in our methods (see Supplementary Table 2 for full-text article exclusion rationale), leading to 15 remaining studies.^16–30^ We included these 15 remaining studies because a risk of bias assessment revealed that all of the studies had a low-to-moderate risk of bias (see Supplementary Table 3). Table 1 briefly summarizes the studies (see Supplementary Table 4 for additional information).

**Table 1.** Characteristics of included studies of the meta-analysis with the individual study data on the MRSA status concordance of the SSTI and nares

Article	Study type	Setting (country)	Participants	Sample processing	True positives	False negatives	False positives	True negatives
Achiam *et al*. 2011	Prospective observational	ED (Canada)	205 Adults	Nares: Culture & PCR SSTI: Culture	14	13	7	171
Acquisto *et al*. 2018	Prospective observational	ED (USA)	116 Adults	Nares: PCR SSTI: Culture	30	22	5	59
Albrecht *et al*. 2015	Case–Control Study	ED (USA)	147 Adults	Nares: Culture SSTI: Culture	33	16	4	94
Alfaro *et al*. 2006	Point prevalence study	Hospital (USA)	30 Children	Nares: Culture SSTI: Not reported	4	10	5	11
Chen *et al*. 2009	Baseline of RCT	Outpatient (USA)	93 Children	Nares: Culture SSTI: Culture	20	44	1	28
Faden *et al*. 2010	Case–Control Study	ED (USA)	60 Children	Nares: Culture SSTI: Culture	5	31	0	24
Frazee *et al*. 2005	Prospective Observational Study	ED (USA)	119 Adults	Nares: Culture SSTI: Culture	21	40	4	54
Gunderson *et al*. 2016	Retrospective Cohort	Hospital (USA)	167 Adults	Nares: PCR SSTI: Culture	36	22	8	101
Hitchcock *et al*. 2023	Retrospective Cohort	Hospital (USA)	300 Adults	Nares: PCR (culture only if PCR indeterminate) SSTI: Culture	35	20	15	230
Jeevannavar *et al*. 2020	Prospective observational	Outpatient (India)	100 Adults & Children	Nares: Culture SSTI: Culture	16	19	9	56
Nurjadi *et al*. 2015	Prospective observational	Outpatient (Netherlands, Spain, Germany, Finland, France, Switzerland, Austria)	310 (Ages not reported)	Nares: Culture SSTI: Culture	14	9	4	283
Pardos de la Gandara *et al*. 2015	Prospective observational	Outpatient (USA)	129 (Ages not reported)	Nares: Culture SSTI: Culture	14	25	5	85
Schleyer *et al*. 2010	Retrospective Cohort	Hospital (USA)	52 Adults	Nares: Culture SSTI: Culture	21	17	0	14
Singh *et al*. 2016	Cross-sectional	Outpatient (USA)	40 Adults	Nares: Culture SSTI: Culture	7	13	1	19
Wananukul *et al*. 2018	Cross-sectional	Hospital (Thailand)	102 Children	Nares: Culture SSTI: Culture	3	0	1	98

We included 1,970 participants in the meta-analysis. An equal number of studies were conducted in the emergency department (5), hospital (5), and outpatient setting (5). Most studies were performed in the United States of America (USA) (11). Demographic reporting was inconsistent among studies. For example, 12 studies^16–18,20–26,29,30^ included information on the sex of their participants (1106 male; 685 female), but some also incorporated non-cultured SSTI cases (ie, patients not in the meta-analysis). Only nine studies^16–18,21,23–25,29,30^ contained information on the sex of patients with both nares and SSTI isolate data (776 male; 459 female). For the remainder of the results, we will discuss the demographic data exclusive to the participants used in the meta-analysis (Supplementary Table 5). The patients included in the meta-analysis had an age range of 1 month to 84 years.^16–18,24,25,28–30^ Abscess/Furuncle was the most reported SSTI (n = 714).^17^ ^,^ ^18^ ^,^ ^21^ ^–^ ^25^ ^,^ ^29^ ^,^ ^30^ Among the 1,051 participants in studies that reported comorbidities,^17,^ ^18^ ^,^ ^22^ ^–^ ^25^ ^,^ ^30^ 275 (26%) had diabetes. Of the 999 participants in studies that reported social history,^16^ ^,^ ^17^ ^,^ ^22^ ^–^ ^24^ ^,^ ^28^ ^,^ ^29^ 60 (6%) were homeless, 150 (15%) were IV drug users, and 26 (3%) resided in an assisted living facility or nursing home.

**Figure 1.**
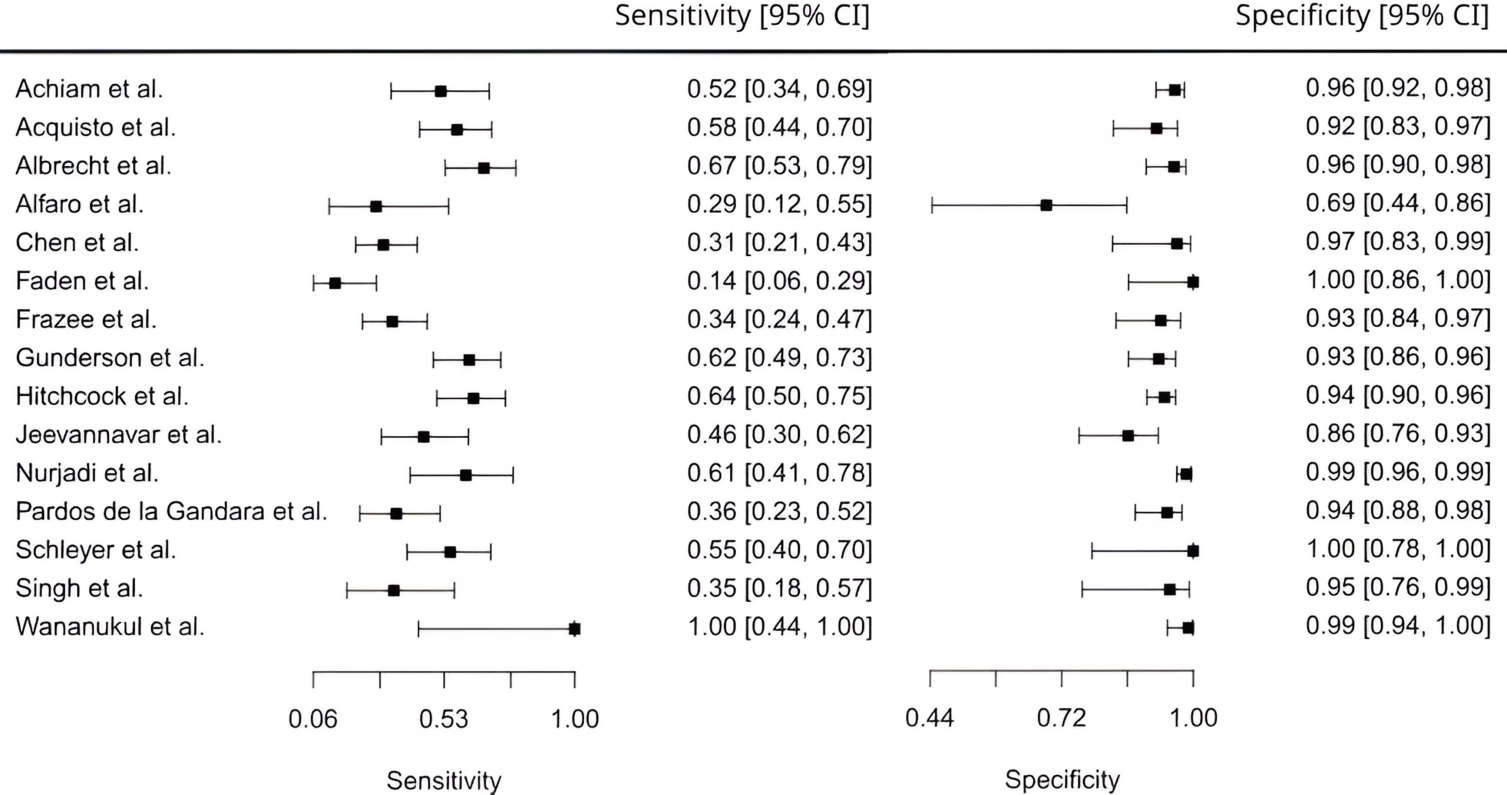
Forest plots of individual sensitivities and specificities with 95% confidence intervals (CIs).

After we incorporated the specificities and sensitivities of the individual studies (Figure 1) into the statistical model, the estimate of the overall specificity was 0.949 (95% CI: 0.920–0.967) and the overall sensitivity was 0.474 (95% CI: 0.383–0.567). The pooled prevalence of MRSA SSTI was 29.1%. Using this prevalence of 29.1%, the positive predictive value (PPV) of MRSA nares swabs for predicting MRSA SSTIs was 0.798 and the negative predictive value (NPV) was 0.815.

Three sub-analyses were performed to assess the heterogeneity of the studies: (1) adult versus pediatric populations, (2) nares isolate analysis with primarily culture versus PCR, and (3) USA versus non-USA studies. The sub-analyses (Table 2) found that screening was more sensitive among adults (0.543 [95% CI: 0.432–0.648]) than among children (0.285 [95% CI: 0.164–0.470]) (median difference of 0.258; *P* < .05). In addition, studies conducted in the USA found a significantly higher MRSA SSTI prevalence (38.8%) than those conducted elsewhere (12.3%; *P* < .01). This difference in baseline prevalence resulted in different predictive values for USA studies (PPV of 0.859 and NPV of 0.741) compared with non-USA studies (PPV of 0.574 and NPV of 0.928).

**Table 2.** Results of sub-analyses assessing whether the subgroups have variable sensitivities and specificities of nasal screening for MRSA to determine if MRSA caused an acute SSTI

Sub-analysis	Comparison groups	Sensitivity [95% CI]	Specificity [95% CI]
Adult versus pediatric samples	Adult (8 studies, n = 1,146)	0.543 [0.432–0.648]^a^	0.947 [0.912–0.971]
	Pediatric (4 studies, n = 285)	0.285 [0.164, –0.470]^a^	0.956 [0.890–0.985]
Culture versus PCR processing of nares samples	Culture (11 studies, n = 1,182)	0.421 [0.320–0.531]	0.954 [0.921–0.980]
	PCR (3 studies, n = 583)	0.611 [0.422–0.769]	0.931 [0.839–0.972]
USA versus non-USA	USA (11 studies, n = 1,253)	0.443 [0.336–0.551]	0.939 [0.903–0.965]
	Non-USA (4 studies, n = 717)	0.574 [0.373–0.766]	0.966 [0.929–0.986]

a Significant (*P* < .05) difference for the sensitivity of adult versus pediatric samples. Other sub-group analyses (eg, culture versus PCR; USA versus non-USA; specificity of adult versus pediatric samples) were not significantly different.

## Discussion

This meta-analysis of 15 studies (n = 1,970)^16^ ^–^ ^30^ revealed that the patient’s MRSA nasal carrier status (ie, carrier or non-carrier) predicted whether MRSA caused the patient’s acute SSTI with an overall specificity of 0.949 and sensitivity of 0.474. This is comparable to diabetic foot infections (specificity = 0.941; sensitivity = 0.417),^5^ which is reassuring for the test’s consistency. Our meta-analysis found a pooled MRSA SSTI prevalence of 29.1% leading to a PPV of 0.798 and an NPV of 0.815. Ideally, screening tools designed to rule out diseases or infections (eg, for antibiotic de-escalation) should have a high NPV. Our meta-analysis found that the NPV of nares MRSA screening was lower for acute SSTIs than for pneumonias (NPV of 0.965 for 10% prevalence),^7^ indicating that screening the nares to predict whether an infection is caused by MRSA is generally less reliable for acute SSTIs than it is for pneumonias. However, the NPV of MRSA nares screening decreases as the MRSA infection prevalence increases. The global baseline MRSA prevalence of SSTIs is 41%,^31^ but can be less than 5% in some countries.^32^ Given the difference in MRSA SSTI prevalence between studies conducted in the USA (38.8%) and those conducted elsewhere (12.3%), our meta-analysis found that the NPV of nasal MRSA screening for patients with acute SSTIs was lower in the USA (0.741) than for studies done elsewhere (0.928). Our sub-analysis examining study location (USA vs non-USA) found that the sensitivities and specificities of MRSA nares screening did not vary by location, indicating that the test performance is similar regardless of location, but the interpretation of the test results (ie, the reliability of a negative test) may differ.

In addition to geography, patient-dependent factors, including history of MRSA infection, IV drug use, diabetes, and HIV/AIDS, can also influence the probability of MRSA SSTIs.^33^ We tried to minimize this type of bias by excluding studies that required certain conditions or treatments as part of the enrollment criteria. However, a significant portion of participants in our meta-analysis had diabetes (26%) or used drugs intravenously (15%), which may have impacted the baseline MRSA SSTI prevalence. We could not analyze the effect of co-morbidities or patient history because demographic data were not reported consistently across studies. However, we were able to analyze differences in pediatric versus adult participants. Interestingly, we found that screening was significantly more sensitive for adults (0.543) than for pediatric participants (0.285), but the specificity did not differ. The reasons underlying this finding are unclear, highlighting the need for continued research into the effect of age on the mechanisms underlying MRSA infections. In addition, the rates of MRSA infections can sometimes differ between pediatric and adult patients,^34^ which would alter the NPV (and therefore the reliability and interpretation) of the MRSA nares screening test for children. Therefore, clinicians should consider the clinical context when using MRSA nares screening to guide management of patients with acute SSTIs.

We also performed a sub-analysis to examine the effect of processing nasal specimens using PCR versus culture. We found no differences in sensitivities or specificities for studies that used PCR versus culture, which aligns with previous literature, where PCR assays for MRSA are comparable to culture detection (sensitivity = 0.976, specificity = 0.949).^35^ Although we incorporated studies with different methods of screening (PCR and culture), the nose was the only screening site in our meta-analysis. Multiple swab sites, particularly the groin and throat, improve detection of MRSA-colonized patients.^36^ Compared with testing multiple body sites, nares-only collections have a sensitivity of 0.68 for MRSA colonization.^36^ However, nares testing is convenient, noninvasive, and widely used,^8^ and we have substantially less data on MRSA colonization at other body sites. Further research is needed to determine the utility of using multiple screening sites for MRSA in clinical practice.

## Supporting information

Bohjanen et al. supplementary material Bohjanen et al. supplementary material

## Data Availability

The data supporting the conclusions of this study are found within the article, supplementary material, and by consulting the works cited.

## Supplementary material

The supplementary material for this article can be found at https://doi.org/10.1017/ice.2025.58.
