# Supplementary material for: Testing for methicillin-resistant Staphylococcus aureus in the anterior nares for antibiotic de-escalation in patients presenting with acute skin and soft tissue infections: systematic review and meta-analysis
Source: Infect Control Hosp Epidemiol. 2025 Apr 16;46(7):716–20. doi: 10.1017/ice.2025.58 (PMC12277076; doi:10.1017/ice.2025.58)
Supplement: Bohjanen et al. supplementary material [file S0899823X25000583sup001.docx]

**Supplementary Table 1: Exact Search Parameters**

| Database | Exact Search | Applicable Filters Used |
| --- | --- | --- |
| PubMed | (MRSA OR "methicillin resistant staphylococcus aureus" OR "methicillin-resistant staphylococcus aureus") AND (skin OR cutaneous OR derm*) AND (SSTI OR "skin and soft tissue infection" OR pyoderma OR "skin infection") AND (Nose OR Nasal OR nares) NOT (Murine[ti]) NOT (Mouse[ti]) NOT (Review[ti]) NOT (Case[ti]) | Include: Human, English  Exclude: None |
| MEDLINE via Ovid | (MRSA OR "methicillin resistant staphylococcus aureus" OR "methicillin-resistant staphylococcus aureus" ) AND (skin OR cutaneous OR derm* ) AND (SSTI OR "skin and soft tissue infection" OR pyoderma OR "skin infection" ) AND (Nose OR Nasal OR nares ) NOT (Murine.ti.) NOT (Mouse.ti.) NOT (Review.ti.) NOT (Case.ti.) | Include: Human, English  Exclude: None |
| Embase via Ovid | (MRSA OR "methicillin resistant staphylococcus aureus" OR "methicillin-resistant staphylococcus aureus" ) AND (skin OR cutaneous OR derm* ) AND (SSTI OR "skin and soft tissue infection" OR pyoderma OR "skin infection" ) AND (Nose OR Nasal OR nares ) NOT (Murine.ti.) NOT (Mouse.ti.) NOT (Review.ti.) NOT (Case.ti.) | Include: Human, English, Article, Letter, Note  Exclude: MEDLINE entries |
| Web of Science | (MRSA OR "methicillin resistant staphylococcus aureus" OR "methicillin-resistant staphylococcus aureus") AND (skin OR cutaneous OR derm*) AND (SSTI OR "skin and soft tissue infection" OR pyoderma OR "skin infection" ) AND (Nose OR Nasal OR nares ) | Include: English, Articles  Exclude: None |
| Scopus | ( mrsa OR "methicillin resistant staphylococcus aureus" OR "methicillin-resistant staphylococcus aureus" ) AND ( skin OR cutaneous OR derm* ) AND ( ssti OR "skin and soft tissue infection" OR pyoderma OR "skin infection" ) AND ( nose OR nasal OR nares ) | Include: English, Human, Article, Letter, Note  Exclude: None |
| Cochrane | (MRSA OR "methicillin resistant staphylococcus aureus" OR "methicillin-resistant staphylococcus aureus" ) AND (skin OR cutaneous OR derm* ) AND (SSTI OR "skin and soft tissue infection" OR pyoderma OR "skin infection" ) AND (Nose OR Nasal OR nares ) | None |

**Data Collection Form:**

Study Type:

Subjects:

Year:

Location:

Participant Inclusion Criteria:

Participant Exclusion Criteria

Sample Collection Methods:

Sample Processing Methods:

Number of participants:

If available, demographic information:

Sex:

Age:

Type of SSTI:

Chronic Diseases:

Living Situation(s):

IVDU:

Hx recent infection:

Hx SSTI:

Hx MRSA:

Hx recent antibiotic use:

Hx recent hospitalization:

MRSA Nares and SSTI Data:

Contingency Table:

|  | | MRSA Wound Culture | |
| --- | --- | --- | --- |
|  |  | Positive | Negative |
| MRSA Nares | Positive |  |  |
|  | Negative |  |  |

**QUADAS-2 Form**

*** Will have 3 signaling questions for each domain. Overall assessment will be the majority answer for the signaling questions. If one domain includes all three answers (yes/no/unclear) with no majority, then overall assessment will be “unclear.”

**DOMAIN 1: PATIENT SELECTION**

- Was a consecutive or random sample of patients enrolled? YES/NO/UNCLEAR
- Did the study avoid inappropriate exclusions? YES/NO/UNCLEAR
- Did the study avoid selecting for a particular patient population (aside from participants with acute SSTI and aside from pediatric vs adult)? YES/NO/UNCLEAR

Bias Assessment: LOW/ HIGH/ UNCLEAR

Concern for Limited Applicability to Meta-Analysis: LOW/ HIGH/ UNCLEAR

**DOMAIN 2: INDEX TEST (Nares MRSA status)**

- If a threshold was used, was it pre-specified? NA/YES/NO/UNCLEAR
- Was the nares isolate collection method the same for all participants? YES/NO/UNCLEAR
- Was the nares isolate processing method the same for all participants? YES/NO/UNCLEAR

Bias Assessment: LOW/ HIGH/ UNCLEAR

Concern for Limited Applicability to Meta-Analysis: LOW/ HIGH/ UNCLEAR

**DOMAIN 3: REFERENCE STANDARD (SSTI lesion MRSA status)**

- Is the reference standard likely to correctly classify the target condition? YES/NO/UNCLEAR
- Was the SSTI isolate collection method the same for all participants? YES/NO/UNCLEAR
- Was the SSTI isolate processing method the same for all participants? YES/NO/UNCLEAR

Bias Assessment: LOW/ HIGH/ UNCLEAR

Concern for Limited Applicability to Meta-Analysis: LOW/ HIGH/ UNCLEAR

**DOMAIN 4: FLOW AND TIMING**

- Did all included SSTI participants undergo anterior nares isolate collection? YES/NO/UNCLEAR
- Did all included SSTI participants undergo SSTI isolate collection? YES/NO/UNCLEAR
- Were the results reported for all nares and SSTI isolates? YES/NO/UNCLEAR

Bias Assessment: LOW/ HIGH/ UNCLEAR

Concern for Limited Applicability to Meta-Analysis: LOW/ HIGH/ UNCLEAR

**Supplementary Figure 1: Flow Diagram of Article Screening and Selection Process**

**Manual Identification**

**Identification of studies via databases**

Records identified from reference searching of included articles (n=414)

Records identified from:

PubMed (n = 74)

Ovid MEDLINE (n = 74)

EMBASE (sans MEDLINE) (n= 43)

Web of Science (n= 124)

Scopus (n= 280)

Cochrane (n= 31)

Records removed *before screening*:

Duplicate records removed (n = 278)

**Identification**

Records included based on title or abstract

(n =106)

Duplicates Removed (n=27)

Records screened

(n = 348)

Records excluded based on title or abstract

(n = 222)

Reports sought for retrieval

(n = 79)

Reports not retrieved

(n =0)

Reports not retrieved

(n = 0)

Reports sought for retrieval

(n = 126)

**Screening**

Reports excluded (see next page)

Reports excluded (see next page)

Reports assessed for eligibility

(n =79)

Reports assessed for eligibility

(n = 126)

Studies included in the review (n = 2)

Studies included in the review

(n = 13)

**Included**

Total number of studies included in the review

(n = 15)

**Supplementary Table 2: Excluded Full-Text Articles (records screened at title/abstract stage not included)**

| Reasons for Exclusion | Articles |
| --- | --- |
| Data was previously published | Fritz et al. 2022 [data found in Mork et al. 2018]; Johnson et al. 2015 [Data found in Ellis et al. 2014]; Lemmens et al. 2011 [data found in Faden et al. 2010] |
| Not human | Loh et al. 2009 [non-human]; Moffitt et al. 2021 [only non-human reported]; Rahman et al. 2018 [only non-human nasal swab]; Zhou et al. 2023 [only non-human nasal swab |
| n<15 | Blum et al. 2022 [n=2]; Creech et al. 2010 [n=5]; Jiménez-Truque et al. 2016 [n=10]; Kazakova et al. 2005 [n=5]; Low et al. 1981 [n=1]; Manzur et al. 2012 [n=10 MRSA SSTI, and an unspecified number of other SSTI]; Masiuk et al. 2010 [n=11]; Miko et al. 2013 [n=10]; Ogura et al. 2021 [n=10]; Shet et al. 2009 [n=10]; Tang et al. 2007 [n=4]; Tinelli et al. 2009 [n=11 sporadic cases] |
| No acute SSTI presentation | Adcock et al. 1998 [colonization only]; Armin et al. 2009; Bhalla et al. 2007; Creech et al. 2005; Frank et al. 2010; Gorowitz et al. 2008; Harbarth et al. 2007; Ho et al. 2008 ["skin lesion" may include chronic, non-infected wounds]; Hussain et al. 2001; Immergluck et al. 2004; Inagawa et al. 2024 [can develop SSTI after admission (hospital-acquired)]; Lucet et al. 2009; Meumann et al. 2016 ["skin lesion" may include chronic, non-infected wounds]; Nakamura et al. 2002; Nilsson et al. 2006; Petry et al. 2020 [can develop SSTI up to 30 days after admission]; Sherertz et al. 1996; Tenover et al. 2008; Wenzel et al. 2008 |
| More than 72 hours between nares and SSTI isolate collections (or unspecified time interval) | Acuna-Villaorduna et al. 2017 [nares screening at admission, but SSTI swab with clinical infection (not necessarily at admission, unspecified time)]; Adeyanju et al. 2022 [Average of 17.5 days, per ST2]; Braga et al. 2023 [up to 30 days]; Burgoon et al. 2022 [unspecified; nares collection not necessarily at admission]; Ellis et al. 2004 [unspecified interval]; Lesens et al. 2007 [unspecified interval]; Mergenhagen et al. 2020 [up to 7 days]; Miller et al. 2012 [up to 21 days]; Mork et al. 2018 [up to 95 days, median of 20 days]; Sligl et al. 2007 [median time = 11 days]; Smith et al. 2018 [up to >1 month]; Stevens et al. 2010 [up to 3.6 years]; Wardyn et al. 2015 [up to multiple months]; Yang et al. 2010 [SSTI Cx within 72 hours of admission, but timeframe for nares collection is ill-defined (sometime after SSTI culture positivity)] |
| Unable to form 2x2 table: No nasal colonization swab | Adam et al. 2009; Barrett et al. 2004; Bhat et al. 2016; Biedenbach et al. 2014; Borgundvaag et al. 2008; Clay et al. 2021; Edelsberg et al. 2009; Faden et al. 2007; Forcade et al. 2011; Fortunov et al. 2006; Freedman et al. 2006; Fridkin et al. 2005; Furtado et al. 2014; Gandhi et al. 2012; Gu et al. 2016; Haran et al. 2019; Henderson et al. 2023; Hochedez et al. 2009; Hudson et al. 2012; Jayakumar et al. 2013; Klein et al. 2019; Klotz et al. 2019; Landrum et al. 2012; Lee et al. 2015; Lee et al. 2016; Liang et al. 2019; Lina et al. 1999; Lipsky et al. 2014; Liu et al. 2009; Liu et al. 2016; Magilner et al. 2008; Malhotra et al. 2012; McCaig et al. 2006; McDonald et al. 2006 [only throat swab]; Meislin et al. 1977; Merritt et al. 2013; Miller et al. 2007; MMWR Morb Mortal Wkly Rep 2003; 52; Mokhtari et al. 2018; Moran et al. 2006; Naimi et al. 2003; Nishijima et al. 2002; Okuma et al. 2002; Patil et al. 2006; Purcell et al. 2005; Ray et al. 2013; Salmanov et al. 2019; Sattler et al. 2002; Shukla et al. 2010 [No nares swabs for MRSA SSTI group]; Stenstrom et al. 2009; Summanen et al. 1995; Talan et al. 2011; Thind et al. 2010; Tomasz et al. 1991; Venniyil et al. 2016 [only nasal swab of non-infected contacts]; Walraven et al. 2012; Williams et al. 2011 |
| Unable to form 2x2 table: No SSTI isolate collection | Fritz et al. 2009; Gasch et al. 2012 [only survey and swabs of chronic ulcers]; Gopal et al. 2007; Kuo et al. 2014 [only survey ]; Oliva et al. 2013; Orellana et al. 2016 [only survey]; Packers et al. 2019 [only survey] |
| Unable to form 2x2 table: Isolates from nares and infection were collected from different people | Crum et al. 2006; Feil et al. 2003; Kenner et al. 2003; Lee et al. 2017; Lo et al. 2010; Mercer et al. 2017; Monaco et al. 2013; Renwick et al. 2008; Sdougkos et al. 2008; Straka et al. 2022; Tang et al. 2011; Trościańczyk et al. 2023 |
| Unable to form 2x2 Table: Other isolates obfuscate nares data | Fritz et al. 2012 [Nares mixed with other sites and contacts data]; Immergluck et al. 2017 [Nares mixed with axillae data]; Kumar et al. 2015 [Nares mixed with throat and groin data]; Pelzek et al. 2018 [Nares data mixed with groin data]; Santosaningsih et al. 2018 [Nares mixed with throat data]; Shaban et al. 2021 [Nares data mixed with axillae and groin data] |
| Unable to form 2x2 table: MRSA status of SSTI not reported | Arhin & Moeck 2017; Chopra et al. 1995; Hidron et al. 2005; Hobbs et al. 2018; Joore et al. 2013; Mithoe et al. 2012 [Two +MRSA Cx, uncertain if from nares vs SSTI]; Namura et al. 1995; Okoye et al. 2019; Taguchi et al. 2010 |
| Unable to form 2x2 table: Non-MRSA SSTI not included/reported | Berla-Kerzhner et al. 2016; Coronado et al. 2007; Ellis et al. 2009; Embil et al. 1994; Evans et al. 2013; Johansson et al. 2007; Millar et al. 2019; Rahimian et al. 2007; Read et al. 2018; Ridgway et al. 2013 [only MRSA SSTI or uncultured clinical diagnosis of SSTI]; Romano et al. 2006 [only MRSA SSTI or uncultured clinical diagnosis of SSTI]; Rosenthal et al. 2006; Suh et al. 1998; Wagenlehner et al. 2007; Zafar et al. 2007 |
| Unable to form 2x2 table: Relationship between MRSA status of nares and SSTI not reported | Ali et al. 2020; Antonov et al. 2015; Artzi et al. 2015; Durupt et al. 2007; Farley et al. 2008; Farley et al. 2013; Ghosh et al. 2022; Iqbal et al. 2018; Kaïret et al. 2017; Krishna et al. 2019; Piper-Jenks et al. 2016; Szumowski et al. 2009; Terpenning et al. 1994; Tobin et al. 2021 |
| Unable to form 2x2 table: Other | Chou et al. 2015 [written data conflicts with table]; Issartel et al. 2005 [2 SSTI/nares pairs have discordant strains, but not necessarily reflective of the MRSA status]; Jones et al. 2014 [Lack nares MRSA status for SSTI patients]; Nagaraju et al. 2004 [99 cases have MRSA status concordance between SSTI and nares, but does not specify number of +MRSA or -MRSA]; Nurjadi et al. 2019 [4 SSTI/nares pairs have discordant strains, but not necessarily reflective of the MRSA status]; Wibbenmeyer et al. 2008 [Relationship between MRSA status of nares and non-MRSA SSTI not reported] |
| Potentially biased inclusion/exclusion criteria | Bar-Meir & Tan 2010 [Excludes patients with any history of MRSA infection/colonization]; Ellis et al. 2014 [hygiene and education interventions]; Emilda et al. 2014 [Excludes patients with any history of MRSA infection/colonization]; Gilbert et al. 2007 [Includes only patients with a history of illicit drug use, homelessness, or incarceration in the prior 6 months]; Kaur et al. 2023 [Excludes non-septic patients]; Millar et al. 2015 [undergoing decolonization interventions]; Nguyen et al. 2005 [hexachlorophene showers or antibiotics]; Sobhy et al. 2012 [Excludes patients with any history of MRSA infection]; Weintrob et al. 2015 [decolonization interventions]; Whitman et al. 2010 [using CHG]; Whitman et al. 2012 [using CHG]; Zanger et al. 2012 [Excludes patients with recent prior SSTI] |

**Supplementary Table 3: Risk of Bias Assessment**

| Article | Patient Selection | | Index Test | | Reference Standard | | Flow and Timing | |
| --- | --- | --- | --- | --- | --- | --- | --- | --- |
|  | Risk of Bias | Applicability Concerns | Risk of Bias | Applicability Concerns | Risk of Bias | Applicability Concerns | Risk of Bias | Applicability Concerns |
| Achiam et al. 2011 | Low | Low | Low | Low | Low | Low | Low | Low |
| Acquisto et al. 2018 | Low | Low | Low | Low | Unclear | Low | Low | Low |
| Albrecht et al. 2018 | Low | Low | Low | Low | Low | Low | Low | Low |
| Alfaro et al. 2006 | Low | Low | Low | Low | Unclear | Low | Low | Low |
| Chen et al. 2009 | Low | Low | Low | Low | Low | Low | Low | Low |
| Faden et al. 2010 | Low | Low | Low | Low | Low | Low | High | Unclear |
| Frazee et al. 2005 | Low | Low | Low | Low | Low | Low | Low | Low |
| Gunderson et al. 2016 | Low | Low | Low | Low | Low | Low | Low | Low |
| Hitchcock et al. 2023 | Unclear | Low | Low | Low | Low | Low | Low | Low |
| Jeevannavar et al. 2020 | Low | Low | Low | Low | Low | Low | Low | Low |
| Nurjadi et al. 2015 | High | Unclear | Low | Low | Low | Low | Low | Low |
| Pardos de la Gandara et al. 2015 | Low | Low | Low | Low | Low | Low | Low | Low |
| Schleyer et al. 2010 | Low | Low | Low | Low | Low | Low | Low | Low |
| Singh et al. 2016 | Unclear | Unclear | Low | Low | Low | Low | High | Unclear |
| Wananukul et al. 2018 | Low | Low | Low | Low | Low | Low | Low | Low |

**Supplementary Table 4: Study Design Variables**

| Article | Study Type | Year(s) Conducted | Location | Setting (# Sites) | Excluded SSTIs | Additional Exclusions | Nasal Swab Brand | SSTI collection | Nares Sample Processing | SSTI Sample Processing |
| --- | --- | --- | --- | --- | --- | --- | --- | --- | --- | --- |
| Achiam et al. 2011 | Prospective Observational | 7/2008-8/2008 | London, Ontario, Canada | ED (3) | Bartholin gland abscess, odontogenic infections, or perianal abscesses | Previous enrollment; Age<18 | Starswabs II (StarplexScientific Inc.); Sterile | Swab of purulent material or point of maximum erythema | Culture with PCR Confirmation (Culture: MRSA Select (BioRad); PCR: In-house multiplex) | Culture (standard techniques and susceptibilities with Vitek 2 system (bioMerieux)) |
| Acquisto et al. 2018 | Prospective Observational | 5/2010-11/2011 | Rochester, NY, USA | ED (1) | Odontogenic infection, Bartholin gland abscess, surgical site infection, animal/human bite | Previous enrollment; Non-English speaking; Wound culture not obtained; Hospital admission required; Age <18 | Swab with liquid Stuart transport media (Copan Diagnostics) | Method not reported | PCR (GeneXpert MRSA nasal screen (Cepheid)) | Culture (standard techniques) |
| Albrecht et al. 2015 | Case-Control Study | 2010-2012 | USA | ED (10) | Study only includes non-perirectal closed skin abscesses, so any other SSTIs are excluded | Currently imprisoned; Residing in long term care facility; Major trauma or critical illness; Currently taking antibiotics; Age<18 | Culturette II (Becton-Dickinson) | Collect purulent material after I&D | Culture (Trypticase soy broth with 6.5% NaCl, mannitol salt agar, then blood agar and susceptibility with cefoxitin disk diffusion) | Culture (Trypticase soy broth with 6.5% NaCl, mannitol salt agar, then blood agar and susceptibility with cefoxitin disk diffusion) |
| Alfaro et al. 2006 | Point Prevalence Study | 2/2005 - 3/2005 | Corpus Christi, TX, USA | Hospital (1) | No SSTI restrictions reported | Nares swab beyond 48 hours of admission | BBL Culture Swab (Becton-Dickinson) | Not reported | Culture (mannitol salt agar, then Mueller-Hinton agar with 4% NaCl and with oxacillin (to test susceptibility)) | Not reported |
| Chen et al. 2009 | Baseline data of RCT | 9/2006-12/2007 | Baltimore, MD, USA | Outpatient (1) | Non-purulent SSTI | Age<6 months or Age ≥18 years | BactiSwab II Culturette with modified Stuart’s medium (Remel) | Swab of purulent material after I&D or if spontaneously draining | Culture (BD-CHROMagar for S. aureus (BBL) with confirmation and susceptibilities using BD Phoenix Automated Microbiology System (BD Diagnostics), then CHROMagar-MRSA (BBL)) | Culture (further details not reported) |
| Faden et al. 2010 | Case-Control Study | 12/2006-3/2008 | Buffalo, NY, USA | ED (1) | Study only includes skin abscesses requiring incision and drainage, so other SSTIs are excluded | Age ≥18 years; Neutropenia; Diverting enterostomies; Patients undergoing emergent operations | Not reported | Collect purulent material after I&D | Culture (sheep blood agar, then gram stain and Staphaurex slide agglutination (Remel) for confirmation; study does not report details on susceptibility testing) | Culture (standard techniques) |
| Frazee et al. 2005 | Prospective Observational Study | 10/2003-2/2004 | Oakland, CA, USA | ED (1) | Odontogenic infections, Bartholin gland abscesses | Previous enrollment; Age<18 | Not reported | Collect using swab or after I&D; no SSTI cultures of pure cellulitis | Culture (Standard techniques, with S. aureus identified by colony morphology, coagulase tests, and catalase tests; susceptibilities with MicroScan Pos Combo 20 multibiochemical substrate panels (Dade Behring Inc.)) | Culture (standard techniques) |
| Gunderson et al. 2016 | Retrospective Cohort | 10/2008-9/2013 | West Haven, CT, USA | Hospital (1) | Non-purulent SSTI; ulcers; surgical wounds; chronic wounds | Previous enrollment; Age <18; Patients with superficial swabs of SSTI | Not reported | Collect purulent material using swab, after I&D, or after needle aspiration | PCR (Xpert MRSA PCR assay (Cepheid)) | Culture (standard techniques) |
| Hitchcock et al. 2023 | Retrospective Cohort | 12/2018-10/2021 | New York City, NY, USA | Hospital (1) | No SSTI restrictions reported | Age<18; MRSA nasal screen and SSTI culture obtained after 48 hours of starting antibiotic; Diagnosis of SSTI not confirmed during admission; MRSA SSTI cultures without available susceptibilities | Not reported | Collect using swab, after I&D, from OR culture, or after aspiration | PCR (if indeterminate, then MRSA culture) | Culture (further details not reported) |
| Jeevannavar et al. 2020 | Prospective Observational | 6/2015-6/2016 | Karnataka, India | Outpatient (1) | No SSTI restrictions reported | Immunosuppression; antibiotics (topical or systemic) in the past week; HIV-positive patients | Not reported | Collect purulent material (unspecified method) | Culture (5% sheep blood agar and MacConkey's agar; identification with morphology and biochemical tests; susceptibilities with Kirby-Bauer disk diffusion method) | Culture (5% sheep blood agar and MacConkey's agar; identification with morphology and biochemical tests; susceptibilities with Kirby-Bauer disk diffusion method) |
| Nurjadi et al. 2015 | Prospective Observational | 5/2011-12/2013 | Netherlands, Spain, Germany, Finland, France, Switzerland, Austria | Outpatient Travel Clinics (13) | Study only includes SSTI that developed while traveling outside of Europe or within 30 days of return, so other SSTIs are excluded | Companion travelers to index patient | Not reported | Swab of lesion/material, sometimes after I&D | Culture (Mannitol salt agar, Columbia agar with 5% sheep blood, then coagulase tests; confirmation with coa gene amplification and susceptibilities with disc diffusion) | Culture (Mannitol salt agar, Columbia agar with 5% sheep blood, then coagulase tests; confirmation with coa gene amplification and susceptibilities with disc diffusion) |
| Pardos de la Gandara et al. 2015 | Prospective Observational | 11/2011-3/2013 | New York City, NY, USA | Outpatient Community Health Centers (6) | No SSTI restrictions reported | None Reported | Swabs with liquid Amies transport medium (Puritan Medical) | Collect using swab or after I&D | Culture (Identification/susceptibilities with MicroScan system (Siemens); confirmation with mannitol-salt agar (Becton Dickinson) then Staphaurex assay (Thermo Fisher Scientific)) | Culture (Identification/susceptibilities with MicroScan system (Siemens); confirmation with mannitol-salt agar (Becton Dickinson) then Staphaurex assay (Thermo Fisher Scientific)) |
| Schleyer et al. 2010 | Retrospective Cohort | 8/2005-12/2005 | Seattle, WA, USA | Hospital (1) | Study only includes SSTI coded as cellulitis and/or abscess, other SSTIs are excluded | Age<18; Admitted to ICU or surgical services | Not reported | Collect using swab or after I&D | Culture (susceptibilities with disk diffusion) | Culture (susceptibilities with disk diffusion) |
| Singh et al. 2016 | Cross-Sectional Observational Study | 7/2012-12/2014 | Fort Benning, GA, USA | Outpatient Troop Medical Clinic (1) | Study only includes purulent abscesses, so other SSTIs are excluded (in particular excludes: chronic cellulitis, deep soft tissue infection, surgical site infection, diabetic foot ulcers, animal or human bite wound, and infection involving the genitals) | Age <18; Vascular insufficiency; Bacteremia; Sepsis; Neutropenia | BD BBL Culture-Swabs (BD Diagnostic) | Swab of purulent material after I&D | Culture (Tryptic soy broth (BD Diagnostics) with 6.5% NaCl; further details not specified) | Culture (Tryptic soy broth (BD Diagnostics) with 6.5% NaCl; further details not specified) |
| Wananukul et al. 2018 | Cross-Sectional Prospective Study | 6/2015 – 3/2016 | Bangkok, Thailand | Hospital (1) | No SSTI restrictions reported | Age ≥15 years; antibiotics (topical or systemic) in past month | Not reported | Swab of lesion/material | Culture (blood agar, susceptibility with cefoxitin disc on Mueller-Hinton agar) | Culture (blood agar, susceptibility with cefoxitin disc on Mueller-Hinton agar) |

**Supplementary Table 5: Demographic Data of Participants included in Meta-Analysis (i.e., those with SSTI and Nares Data)**

| Article | Participants with SSTI and nares data (n) | Sex (n (%)) | Age in years (mean (SD)) or (mean [range]) | SSTI Types* (n) | Chronic Conditions (n) | Social History (n) | Infectious Exposures and History (n) |
| --- | --- | --- | --- | --- | --- | --- | --- |
| Achiam et al. 2011 | 205 | Male: 107 (52%)  Female: 98 (48%) | 45.3 (17.9) | Not Reported | Not Reported | Homeless: 10  Incarcerated (past year): 9  IV drug use: 40 | Hospitalization (past year): 43  Abscess (past year): 82  Cellulitis (past year): 52  Wound infection (past year): 66  Antibiotic use (past 3 months): 79 |
| Acquisto et al. 2018 | 116 | Male: 69 (59%)  Female: 47 (41%) | 32.8 (11.4) | Abscess: 72  Cellulitis: 24  Abscess/cellulitis mix: 19  Ulcer infection (acute): 2  Paronychia: 1 | Diabetes: 17  Immunosuppression: 2  Hemodialysis: 2 | Living situation:  -Treatment facility: 3  -Nursing home: 1  -Dormitory: 3  -Homeless: 3  IV drug use: 13 | Hospitalization (past year): 30  SSTI (past year): 53  Antibiotic use (past 3 months) :32 |
| Albrecht et al. 2015 | 147 | Male: 85 (59%)  Female: 60 (41%)  Unknown: 2 | 38 [18 to 82] | Abscess: 147 | Diabetes: 12  Chronic liver failure: 1  Atopic dermatitis: 9 | Not Reported | SSTI (past 6 months): 46  MRSA (ever): 15 |
| Alfaro et al. 2006 | 30 | No separate demographic data reported for subset with SSTI and nares data | | | | | |
| Chen et al. 2009 | 93 | No separate demographic data reported for subset with SSTI and nares data | | | | | |
| Faden et al. 2010 | 60 | Male: 26 (43%)  Female: 34 (57%) | Not Reported | Abscess/Furuncle: 60 | Not Reported | Not Reported | Previous abscess: 16 |
| Frazee et al. 2005 | 119 | Not Reported | Not Reported | Abscess: 86  Infected ulcer (acute): 14  Infected wound (acute): 10 | Diabetes (insulin-dependent): 7 | Homeless: 23  Incarcerated (past year): 31  IV drug use: 33 | Hospitalization (past year): 32  Abscess (past year): 44  Antibiotic use (past 3 months): 44 |
| Gunderson et al. 2016 | 167 | Male: 160 (96%)  Female: 7 (4%) | Not Reported | Abscess: 144  Cellulitis: 15 | Diabetes: 92  Immunosuppression: 15  Cancer: 12  Peripheral artery disease: 8  ESRD: 6  Cirrhosis: 4  HIV: 3 | Alcohol use disorder: 17  IV drug use: 10  Assisted living: 6 | Antibiotic use (past 30 days): 64 |
| Hitchcock et al. 2023 | 300 | Male: 181 (60%)  Female: 119 (40%) | 56 (15.3) | Abscess: 108  Cellulitis: 50  Infected ulcer (acute): 41  Infected surgical site: 18  Infected burn: 10 | Diabetes: 138  Dialysis: 10 | Nursing facility: 19  Homeless: 5  IV drug use: 31 | Not Reported. |
| Jeevannavar et al. 2020 | 100 | Male: 64 (64%)  Female: 36 (36%) | 28.5 (19) | Abscess/Furuncle: 57  Folliculitis: 17  Impetigo: 15  Paronychia: 5  Carbuncle: 4  Erysipelas: 2 | Diabetes: 9 | Not Reported | Not Reported |
| Nurjadi et al. 2015 | 310 | No separate demographic data reported for subset with SSTI and nares data | | | | | |
| Pardos de la Gandara et al. 2015 | 129 | Not Reported | Not Reported | Not Reported | Not Reported | Not Reported | Not Reported |
| Schleyer et al. 2010 | 52 | Not Reported | [21 to 84] | Not Reported | Not Reported | Homeless: 19  Incarcerated (current): 6  IV drug use: 23 | History of MRSA: 19 |
| Singh et al. 2016 | 40 | Male: 40 (100%)  Female: 0 (0%) | [18 to 28] | Abscess: 40 | Not Reported | US Army soldiers (infantry training): 40 | Not Reported |
| Wananukul et al. 2018 | 102 | Male: 44 (43%)  Female: 58 (57%) | 6.1 (5) | Secondary bacterial infection: 81  Impetigo: 14  Wound infection: 3  Folliculitis: 2  Ecthyma: 1  Paronychia: 1 | Atopic Dermatitis: 48 | Not Reported | Not Reported |

*Some patients presented with multiple types of SSTI. Some studies only reported certain infections (remaining cases were other/non-specified or not reported)

References for Supplementary Table 2:

1. Acuna-Villaorduna C, Branch-Elliman W, Strymish J, Gupta K. Active identification of patients who are methicillin-resistant Staphylococcus aureus colonized is not associated with longer duration of vancomycin therapy. Am J Infect Control. 2017;45(10):1081-1085. doi:10.1016/j.ajic.2017.04.011
2. Adam HJ, Allen VG, Currie A, et al. Community-associated methicillin-resistant Staphylococcus aureus: prevalence in skin and soft tissue infections at emergency departments in the Greater Toronto Area and associated risk factors. CJEM 2009; 11:439–446.
3. Adcock PM, Pastor P, Medley F, Patterson JE, Murphy TV. Methicillin resistant Staphylococcus aureus in two child care centers. JID. 1998;178: 577–580
4. Adeyanju A, Schaumburg F, Onayade A, et al. Local epidemiology of nosocomial staphylococcus aureus infection in a Nigerian university teaching hospital. Antibiotics (Basel). 2022;11(10):1372. Published 2022 Oct 7. doi:10.3390/antibiotics11101372
5. Ali JF, Al-Saadi ZN, and Saeed YS. Spa gene polymorphism based molecular typing of clinical MRSA strains isolated from Wasit City/Iraq. International Journal of Pharmaceutical Research. 2020.
6. Antonov NK, Garzon MC, Morel KD, Whittier S, Planet PJ, Lauren CT. High prevalence of mupirocin resistance in Staphylococcus aureus isolates from a pediatric population [published correction appears in Antimicrob Agents Chemother. 2015 Nov;59(11):7158. doi: 10.1128/AAC.02220-15.]. Antimicrob Agents Chemother. 2015;59(6):3350-3356. doi:10.1128/AAC.00079-15
7. Arhin FF, Moeck G. Assessment of the potential for oritavancin MIC changes among Staphylococcus aureus nasal carriage isolates following systemic oritavancin treatment in a phase 2 study in patients with acute bacterial skin and skin-structure infections. J Glob Antimicrob Resist. 2017;9:8-9. doi:10.1016/j.jgar.2017.01.003
8. Armin S, Karimi A, Fallah F, Fahimzad A, and Kiomarci A. Methicillin-resistant Staphylococcus aureus: A phantom or true menace in our neonates? Journal of Pediatric Infectious Diseases, 04, 261 - 265.. “Methicillin-resistant Staphylococcus aureus: A phantom or true menace in our neonates?” Journal of Pediatric Infectious Diseases 04 (2009): 261 - 265.
9. Artzi O, Sinai M, Solomon M, Schwartz E. Recurrent furunculosis in returning travelers: newly defined entity. J Travel Med. 2015;22(1):21-25. doi:10.1111/jtm.12151
10. Bar-Meir M, Tan TQ. Staphylococcus aureus skin and soft tissue infections: can we anticipate the culture result?. Clin Pediatr (Phila). 2010;49(5):432-438. doi:10.1177/0009922809350496
11. Barrett TW, Moran GJ. Methicillin-resistant Staphylococcus aureus infections among competitive sports participantsdColorado, Indiana, Pennsylvania, and Los Angeles County, 2000-2003. Ann Emerg Med. 2004;43:43-45.
12. Berla-Kerzhner E, Biber A, Parizade M, et al. Clinical outcomes and treatment approach for community-associated methicillinresistant Staphylococcus aureus (CA-MRSA) infections in Israel. Eur J Clin Microbiol Infect Dis 2016; 36: 153-62.
13. Bhalla A, Aron DC, Donskey CJ. Staphylococcus aureus intestinal colonization is associated with increased frequency of S aureus on skin of hospitalized patients. BMC Infect Dis. 2007;7:105
14. Bhat YJ, Hassan I, Bashir S, et al. Clinicobacteriological profile of primary pyodermas in Kashmir: a hospital-based study. J R Coll Physicians Edinb. 2016; 46: 8-13.
15. Biedenbach DJ, Bouchillon SK, Johnson SA, Hoban DJ, Hackel M. Susceptibility of Staphylococcus aureus to topical agents in the United States: a sentinel study. Clin Ther. 2014;36(6):953-960. doi:10.1016/j.clinthera.2014.04.003
16. Blum FC, Whitmire JM, Bennett JW, et al. Nasal microbiota evolution within the congregate setting imposed by military training. Sci Rep. 2022;12(1):11492. Published 2022 Jul 7. doi:10.1038/s41598-022-15059-z
17. Borgundvaag B, Katz K, Allen V, et al. Prevalence of CA-MRSA in purulent skin and soft tissue infections in patients presenting at emergency departments in the greater Toronto area. CJEM 2008; 10:259.
18. Braga S, Rajapakse N, Heyliger J, Dierkhising R, Dinnes L. Pediatric utilization of methicillin-resistant Staphylococcus aureus nasal swabs for antimicrobial stewardship. Pediatr Infect Dis J. 2023;42(12):e466-e469. doi:10.1097/INF.0000000000004129
19. Burgoon R, Weeda E, Mediwala K, Raux B. Clinical utility of negative methicillin-resistant Staphylococcus aureus (MRSA) nasal surveillance swabs in skin and skin structure infections. Am J Infect Control. 2021(21):941-946. doi:10.1016/j. ajic.2021.12.005.
20. Chopra A, Purl R, Mittal RR. Correlation of isolates from pyoderma and carrier sites. Indian J Dermatol Venereol Leprol. 1995; 61: 273–5.
21. Chou YH, Lee MS, Lin RY, Wu CY. Risk factors for methicillin-resistant Staphylococcus aureus skin and soft-tissue infections in outpatients in Taiwan. Epidemiol Infect. 2015;143(4):749-753. doi:10.1017/S0950268814001642
22. Clay TB, Orwig KW, Stevens RA, et al. Correlation of MRSA polymerase chain reaction (PCR) wound swab testing and wound cultures in skin and soft tissue infections. Diagn Microbiol Infect Dis. 2021;100(4):115389. doi:10.1016/j. diagmicrobio.2021.115389.
23. Coronado F, Nicholas JA, Wallace BJ, et al. Community-associated methicillin-resistant Staphylococcus aureus skin infections in a religious community. Epidemiol Infect. 2007;135(3):492-501. doi:10.1017/S0950268806006960
24. Creech CB, Kernodle DS, Alsentzer A, et al. Increasing rates of nasal carriage of methicillin-resistant Staphylococcus aureus in healthy children. Pediatr Infect Dis J. 2005;24:617– 621.
25. Creech CB, Saye E, McKenna BD, et al. One-year surveillance of methicillin-resistant Staphylococcus aureus nasal colonization and skin and soft tissue infections in collegiate athletes. Arch Pediatr Adolesc Med. 2010;164(7):615-620. doi:10.1001/archpediatrics.2010.93
26. Crum NF, Lee RU, Thornton SA, et al. 2006. Fifteen-year study of the changing epidemiology of methicillin-resistant Staphylococcus aureus.AmJ Med 119:943–951. http://dx.doi.org/10.1016/j.amjmed.2006.01.004.
27. Durupt F, Mayor L, Bes M, et al. Prevalence of Staphylococcus aureus toxins and nasal carriage in furuncles and impetigo. Br J Dermatol. 2007;157(6):1161-1167. doi:10.1111/j.1365-2133.2007.08197.x
28. Edelsberg J, Taneja C, Zervos M, et al. Trends in US hospital admissions for skin and soft tissue infections. Emerg Infect Dis 2009;15:1516-8.
29. Ellis MW, Hospenthal DR, Dooley DP, et al. Natural history of community-acquired methicillin-resistant Staphylococcus aureus colonization and infection in soldiers. Clin Infect Dis. 2004;39:971–979.
30. Ellis MW, Griffith ME, Jorgensen JH, Hospenthal DR, Mende K, Patterson JE. Presence and molecular epidemiology of virulence factors in methicillin-resistant Staphylococcus aureus strains colonizing and infecting soldiers. J Clin Microbiol. 2009;47(4):940-945. doi:10.1128/JCM.02352-08
31. Ellis MW, Schlett CD, Millar EV, et al. Prevalence of nasal colonization and strain concordance in patients with community-associated Staphylococcus aureus skin and soft-tissue infections. Infect Control Hosp Epidemiol. 2014;35(10):1251-1256. doi:10.1086/678060
32. Embil J, Ramotar K, Romance L, Alfa M, Conly J, Cronk S, et al. Methicillinresistant Staphylococcus aureus in tertiary care institutions on the Canadian Prairies 1990–1992. Infect Control Hosp Epidemiol 1994; 15:646–651.
33. Emilda JK, Shenoy SM, Chakrapani M, Kumar P, Bhat KG. Clinical spectrum and antimicrobial resistance pattern of skin and soft tissue infections caused by community acquired-methicillin resistant Staphylococcus aureus. Indian J Dermatol Venereol Leprol. 2014;80(6):539-540. doi:10.4103/0378-6323.144178
34. Evans ME, Kralovic SM, Simbartl LA, et al. Prevention of methicillin-resistant Staphylococcus aureus infections in spinal cord injury units. Am J Infect Control. 2013;41(5):422-426. doi:10.1016/j.ajic.2012.06.006
35. Faden H, Rose R, Lesse A, Hollands C, Dryja D, Glick PL. Clinical and molecular characteristics of staphylococcal skin abscesses in children. J Pediatr. 2007;151(6):700 –703
36. Farley JE, Ross T, Stamper P, Baucom S, Larson E, Carroll KC. Prevalence, risk factors, and molecular epidemiology of methicillin-resistant Staphylococcus aureus among newly arrested men in Baltimore, Maryland. Am J Infect Control. 2008;36(9):644-650. doi:10.1016/j.ajic.2008.05.005
37. Farley JE, Ross T, Krall J, et al. Prevalence, risk factors, and molecular epidemiology of methicillin-resistant Staphylococcus aureus nasal and axillary colonization among psychiatric patients on admission to an academic medical center. Am J Infect Control. 2013;41(3):199-203. doi:10.1016/j.ajic.2012.03.028
38. Feil EJ, Cooper JE, Grundmann H, et al. How clonal is Staphylococcus aureus? J Bacteriol 2003;185:3307–3316. http://dx.doi.org/10.1128/JB.185.11.3307-3316.2003.
39. Forcade NA, Parchman ML, Jorgensen JH, et al. Prevalence, severity, and treatment of community-acquired methicillin-resistant Staphylococcus aureus (CA-MRSA) skin and soft tissue infections in 10 medical clinics in Texas: a South Texas Ambulatory Research Network (STARNet) study. J Am Board Fam Med. 2011; 24: 543-50.
40. Fortunov RM, Hulten KG, Hammerman WA, Mason EO, Kaplan SL. Community-acquired Staphylococcus aureus infections in term and near-term previously healthy neonates. Pediatrics. 2006;118(3):874 – 881.
41. Frank DN, Feazel LM, Bessesen MT, Price CS, Janoff EN, Pace NR. The human nasal microbiota and Staphylococcus aureus carriage. PLoS One 2010;5:e10598. http://dx.doi.org/10.1371/journal.pone.0010598.
42. Freedman DO, Weld LH, Kozarsky PE, et al. Spectrum of disease and relation to place of exposure among ill returned travelers. N Engl J Med 2006;354:119–30.
43. Fridkin S, Hageman JC, Morrison M, et al. Methicillin-resistant Staphylococcus aureus disease in three communities. N Engl J Med 2005; 352:1436–1444.
44. Fritz SA, Epplin EK, Garbutt J, Storch GA. Skin infection in children colonized with community-associated methicillin-resistant Staphylococcus aureus. J Infect. 2009;59(6):394-401. doi:10.1016/j.jinf.2009.09.001
45. Fritz SA, Hogan PG, Hayek G, et al. Staphylococcus aureus colonization in children with community-associated Staphylococcus aureus skin infections and their household contacts. Arch Pediatr Adolesc Med. 2012;166(6):551-557. doi:10.1001/archpediatrics.2011.900
46. Fritz SA, Wylie TN, Gula H, et al. Longitudinal dynamics of skin bacterial communities in the context of Staphylococcus aureus decolonization. Microbiol Spectr. 2022;10(2):e0267221. doi:10.1128/spectrum.02672-21
47. Furtado S, Bhat RM, Rekha B, et al. The clinical spectrum and antibiotic sensitivity patterns of staphylococcal pyodermas in the community and hospital. Indian J Dermatol. 2014;59:143-50.
48. Gandhi S, Ojha AK, Ranjan KP, et al. Clinical and bacteriological aspects of pyoderma. North Am J Med Sci. 2012; 4: 492–5.
49. Gasch O, Hornero A, Domínguez MA, et al. Methicillin-susceptible Staphylococcus aureus clone related to the early pandemic phage type 80/81 causing an outbreak among residents of three occupational centres in Barcelona, Spain. Clin Microbiol Infect. 2012;18(7):662-667. doi:10.1111/j.1469-0691.2011.03663.x
50. Ghosh S, Sengupta M, Sarkar S, et al. Bacteriologic profile along with antimicrobial susceptibility pattern of pediatric pyoderma in Eastern India. Cureus. 2022;14(6):e25716. Published 2022 Jun 7. doi:10.7759/cureus.25716
51. Gilbert M, Macdonald J, Louie M, et al. Prevalence of USA300 colonization or infection and associated variables during an outbreak of community-associated methicillin-resistant Staphylococcus aureus in a marginalized urban population. Can J Infect Dis Med Microbiol. 2007;18(6):357-362. doi:10.1155/2007/597123
52. Gopal Rao G, Michalczyk P, Nayeem N, et al. Prevalence and risk factors for meticillinresistant Staphylococcus aureus in adult emergency admissions--a case for screening all patients? J Hosp Infect 2007;66:15–21.
53. Gorwitz R, Kruszon-Moran D, McAllister S, et al. Changes in the prevalence of nasal colonization with Staphylococcus aureus in the United States, 2001–2004. J Infect Dis. 2008;197:1226 –1234.
54. Gu FF, Chen Y, Dong DP, et al. Molecular Epidemiology of Staphylococcus aureus among Patients with Skin and Soft Tissue Infections in Two Chinese Hospitals. Chin Med J. 2016; 129: 2319-24.
55. Haran J, Wilsterman E, Zeoli T, Goulding M, McLendon E, Clark M. Deviating from IDSA treatment guidelines for non-purulent skin infections increases the risk of treatment failure in emergency department patients. Epidemiol Infect. 2019;147:e68. doi:10.1017/S0950268818003291.
56. Harbarth S, Schrenzel J, Renzi G, Akakpo C, Ricou B. Is throat screening necessary to detect methicillin-resistant Staphylococcus aureus colonization in patients upon admission to an intensive care unit? J Clin Microbiol 2007;45:1072–1073. http://dx.doi.org/10.1128/JCM.02121-06.
57. Henderson KL, Saei A, Freeman R, et al. Intermittent point prevalence surveys on healthcare-associated infections, 2011 and 2016, in England: what are the surveillance and intervention priorities?. J Hosp Infect. 2023;140:24-33. doi:10.1016/j.jhin.2023.07.015
58. Hidron AI, Kourbatova EV, Halvosa JS, et al. Risk factors for colonization with methicillin-resistant Staphylococcus aureus (MRSA) in patients admitted to an urban hospital: Emergence of community-associated MRSA nasal carriage. Clinical Infectious Diseases 2005;41:159–66.
59. Ho PL, Lai EL, Chow KH, Chow LS, Yuen KY, Yung RW. Molecular epidemiology of methicillin-resistant Staphylococcus aureus in residential care homes for the elderly in Hong Kong. Diagn Microbiol Infect Dis. 2008;61(2):135-142. doi:10.1016/j.diagmicrobio.2007.12.017
60. Hobbs MR, Grant CC, Thomas MG, et al. Staphylococcus aureus colonisation and its relationship with skin and soft tissue infection in New Zealand children. Eur J Clin Microbiol Infect Dis. 2018;37(10):2001-2010. doi:10.1007/s10096-018-3336-1
61. Hochedez P, Canestri A, Lecso M, Valin N, Bricaire F, Caumes E. Skin and soft tissue infections in returning travelers. Am J Trop Med Hyg 2009;80:431 – 4.
62. Hudson LO, Murphy CR, Spratt BG, et al. Differences in methicillin-resistant Staphylococcus aureus strains isolated from pediatric and adult patients from hospitals in a large county in California. J Clin Microbiol 2012;50:573–579. http://dx.doi.org/10.1128 /JCM.05336-11.
63. Hussain FM, Boyle-Vavra S, Daum RS. Community-acquired methicillin-resistant Staphylococcus aureus colonization in healthy children attending an outpatient pediatric clinic. Pediatr Infect Dis J. 2001;20: 763–767.
64. Immergluck LC, Kanungo S, Schwartz A, et al. Prevalence of Streptococcus pneumoniae and Staphylococcus aureus nasopharyngeal colonization in healthy children in the United States. Epidemiol Infect. 2004; 132:159 –166.
65. Immergluck LC, Jain S, Ray SM, et al. Risk of skin and soft tissue infections among children found to be Staphylococcus aureus MRSA USA300 carriers. West J Emerg Med. 2017;18(2):201-212. doi:10.5811/westjem.2016.10.30483
66. Inagawa T, Hisatsune J, Kutsuno S, et al. Genomic characterization of Staphylococcus aureus isolated from patients admitted to intensive care units of a tertiary care hospital: epidemiological risk of nasal carriage of virulent clone during admission. Microbiol Spectr. 2024;12(6):e0295023. doi:10.1128/spectrum.02950-23
67. Iqbal MS, Saleem Y, Ansari F, et al. Staphylococcus aureus carrying lukS/F Panton-Valentine Leukocidin (PVL) toxin genes in hospitals of Lahore city. J Infect Dev Ctries. 2018;12(9):720-725. Published 2018 Sep 30. doi:10.3855/jidc.9633
68. Issartel B, Tristan A, Lechevallier S, et al. Frequent carriage of Panton-Valentine leucocidin genes by Staphylococcus aureus isolates from surgically drained abscesses. J Clin Microbiol. 2005;43(7):3203-3207. doi:10.1128/JCM.43.7.3203-3207.2005
69. Jayakumar S, Meerabai M, Shameem Banu AS, et al. Prevalence of high and low level mupirocin resistance among staphylococcal isolates from skin infection in a tertiary care hospital. J Clin Diagn Res. 2013;7(2):238-242. doi:10.7860/JCDR/2013/4694.2736
70. Piper-Jenks N, Pardos de la Gandara M, D'Orazio BM, et al. Differences in prevalence of community-associated MRSA and MSSA among U.S. and non-U.S. born populations in six New York Community Health Centers. Travel Med Infect Dis. 2016;14(6):551-560. doi:10.1016/j.tmaid.2016.10.003
71. Jiménez-Truque N, Saye EJ, Soper N, et al. Longitudinal assessment of colonization with Staphylococcus aureus in healthy collegiate athletes. J Pediatric Infect Dis Soc. 2016;5(2):105-113. doi:10.1093/jpids/piu108
72. Johansson PJ, Gustafsson EB, Ringberg H. High prevalence of MRSA in household contacts. Scand J Infect Dis 2007; 39:764 –768. http://dx.doi .org/10.1080/00365540701302501.
73. Johnson RC, Ellis MW, Lanier JB, Schlett CD, Cui T, Merrell DS. Correlation between nasal microbiome composition and remote purulent skin and soft tissue infections. Infect Immun 2015; 83:802– 811. http:// dx.doi.org/10.1128/IAI.02664-14.
74. Jones M, Huttner B, Leecaster M, et al. Does universal active MRSA surveillance influence anti-MRSA antibiotic use? a retrospective analysis of treatment of patients admitted with suspicion of infection at Veterans Affairs Medical Centers between 2005 and 2010. J Antimicrob Chemother. 2014;69(12):3401-3408. doi:10.1093/jac/dku299.
75. Joore IK, van Rooijen MS, Schim van der Loeff MF, et al. Low prevalence of methicillin-resistant Staphylococcus aureus among men who have sex with men attending an STI clinic in Amsterdam: a cross-sectional study. BMJ Open. 2013;3(3):e002505. Published 2013 Mar 5. doi:10.1136/bmjopen-2012-002505
76. Kaïret K, Ho E, Van Kerkhoven D, et al. USA300, A strain of community-associated methicillin-resistant Staphylococcus aureus, crossing Belgium's borders: outbreak of skin and soft tissue infections in a hospital in Belgium. Eur J Clin Microbiol Infect Dis. 2017;36(5):905-909. doi:10.1007/s10096-016-2883-6
77. Kaur K, Khalil S, Singh NP, Dewan P, Gupta P, Shah D. Antibiotic susceptibility, carrier state and predictors of outcome of Staphylococcus aureus infections in hospitalized children. Indian Pediatr. 2023;60(1):49-53.
78. Kazakova SV, Hageman JC, Matava M, et al. A clone of methicillin-resistant Staphylococcus aureus among professional football players. N Engl J Med 2005;352: 468 – 475. http://dx.doi.org/10.1056/NEJMoa042859
79. Kenner J, O’Connor T, Piantanida N, et al. Rates of carriage of methicillin-resistant and methicillin-susceptible Staphylococcus aureus in an outpatient population. Infect Control Hosp Epidemiol 2003;24:439 – 444. http:// dx.doi.org/10.1086/502229.
80. Klein S, Menz MD, Zanger P, Heeg K, Nurjadi D. Increase in the prevalence of Panton-Valentine leukocidin and clonal shift in community-onset methicillin-resistant Staphylococcus aureus causing skin and soft-tissue infections in the Rhine-Neckar Region, Germany, 2012-2016. Int J Antimicrob Agents. 2019;53(3):261-267. doi:10.1016/j.ijantimicag.2018.10.026
81. Klotz C, Courjon J, Michelangeli C, Demonchy E, Ruimy R, Roger PM. Adherence to antibiotic guidelines for erysipelas or cellulitis is associated with a favorable outcome. Eur J Clin Microbiol Infect Dis. 2019;38(4):703-709. doi:10.1007/s10096-019-03490-6
82. Krishna, PVM, Reddy VS, Kumar VP, and Suresh P. "Antibiotic Susceptibility Pattern of Staphylococcus aureus and Methicillin–Resistant Staphylococcus aureus Isolated from Various Clinical Specimens in a Tertiary Care Teaching Hospital, Pondicherry." EXECUTIVE EDITOR 10.2 (2019): 208.
83. Kumar N, David MZ, Boyle-Vavra S, Sieth J, Daum RS. High Staphylococcus aureus colonization prevalence among patients with skin and soft tissue infections and controls in an urban emergency department. J Clin Microbiol. 2015;53(3):810-815. doi:10.1128/JCM.03221-14
84. Kuo CY, Huang YC, Huang DT, et al. Prevalence and molecular characterization of Staphylococcus aureus colonization among neonatal intensive care units in Taiwan. Neonatology. 2014;105(2):142-148. doi:10.1159/000356733
85. Landrum ML, Neumann C, Cook C, et al. Epidemiology of Staphylococcus aureus blood and skin and soft tissue infections in the US military health system, 2005–2010. JAMA 2012;308:50 –59. http://dx.doi.org/10.1001/ jama.2012.7139.
86. Lee C, Tsai H, Kunin C, Lee S, Chen Y. Clinical and microbiological characteristics of purulent and non-purulent cellulitis in hospitalized Taiwanese adults in the era of community-associated methicillin-resistant Staphylococcus aureus. BMC Infect Dis. 2015;15:311. doi:10.1186/s12879015-1064-z
87. Lee GC, Hall RG, Boyd NK, et al. Predictors of community associated Staphylococcus aureus, methicillin-resistant and methicillin susceptible Staphylococcus aureus skin and soft tissue infections in primary-care settings. Epidemiol Infect. 2016;144: 3198-3204.
88. Lee GC, Dallas SD, Wang Y, et al. Emerging multidrug resistance in community-associated Staphylococcus aureus involved in skin and soft tissue infections and nasal colonization. J Antimicrob Chemother. 2017;72(9):2461-2468. doi:10.1093/jac/dkx200
89. Lemmens N, van Wamel W, Snijders S, Lesse AJ, Faden H, van Belkum A. Genomic comparisons of USA300 Staphylococcus aureus colonizating the nose and rectum of children with skin abscesses. Microb Pathog. 2011;50(3-4):192-199. doi:10.1016/j.micpath.2010.12.006
90. Lesens O, Haus-Cheymol R, Dubrous P, et al. Methicillin-susceptible, doxycycline-resistant Staphylococcus aureus, Côte d'Ivoire. Emerg Infect Dis. 2007;13(3):488-490. doi:10.3201/eid1303.060729
91. Liang Y, Tu C, Tan C, et al. Antimicrobial resistance, virulence genes profiling and molecular relatedness of methicillin-resistant Staphylococcus aureus strains isolated from hospitalized patients in Guangdong Province, China. Infect Drug Resist. 2019;12:447-459. Published 2019 Feb 25. doi:10.2147/IDR.S192611
92. Lina G, Piemont Y, Godail-Gamot F, et al. Involvement of PantonValentine Leukocidin producing Staphylococcus aureus primary skin infections and pneumonia. Clin Infect Dis. 1999;29:1128 –1132
93. Lipsky BA, Napolitano LM, Moran GJ, et al. Economic outcomes of inappropriate initial antibiotic treatment for complicated skin and soft tissue infections: a multicenter prospective observational study. Diagn Microbiol Infect Dis. 2014;79(2):266-272. doi:10.1016/j.diagmicrobio.2014.02.013.
94. Liu Y, Kong F, Zhang X, Brown M, Ma L, Yang Y. Antimicrobial susceptibility of Staphylococcus aureus isolated from children with impetigo in China from 2003 to 2007 shows community-associated methicillin-resistant Staphylococcus aureus to be uncommon and heterogeneous. Br J Dermatol 2009; 161: 1347-50.
95. Liu Y, Xu Z, Yang Z, et al. Characterization of community-associated Staphylococcus aureus from skin and soft-tissue infections: a multicenter study in China. Emerg Microbes Infect. 2016;5: e127.
96. Lo WT, Wang SR, Tseng MH, Huang CF, Chen SJ, Wang CC. Comparative molecular analysis of meticillin-resistant Staphylococcus aureus isolates from children with atopic dermatitis and healthy subjects in Taiwan. Br J Dermatol. 2010;162(5):1110-1116. doi:10.1111/j.1365-2133.2010.09679.x
97. Loh JV, Percival SL, Woods EJ, Williams NJ, Cochrane CA. Silver resistance in MRSA isolated from wound and nasal sources in humans and animals. Int Wound J. 2009;6(1):32-38. doi:10.1111/j.1742-481X.2008.00563.x
98. Low DE, Garcia M, Callery S, Milne P, Devlin HR, Campbell I. Methicillinresistant Staphylococcus aureus- Ontario. Can Dis Wkly Rep 1981; 7:249–250.
99. Lucet JC, Paoletti X, Demontpion C, et al. Carriage of methicillinresistant Staphylococcus aureus in home care settings: prevalence, duration, and transmission to household members. Arch Intern Med 2009; 169:1372–8.
100. Magilner D, Byerly MM, Cline DM. The prevalence of community-acquired methicillinresistant Staphylococcus aureus (CA-MRSA) in skin abscesses presenting to the pediatric emergency department. N C Med J 2008;69:351–4.
101. Malhotra SK, Malhotra S, Dhaliwal GS, et al. Bacteriological study of pyodermas in a tertiary care dermatological center. Indian J Dermatol. 2012;57: 358–61. 18.
102. Manzur A, De Gopegui ER, Dominguez M, et al. Clinical significance of methicillin-resistant Staphylococcus aureus colonization in residents in community long-term-care facilities in Spain. Epidemiol Infect. 2012;140(3):400-406. doi:10.1017/S0950268811000641
103. Straka M, Hubenakova Z, Lichvarikova A, et al. Susceptibility of Staphylococcus aureus strains to commercial therapeutic phage preparations. Bratisl Lek Listy. 2022;123(10):724-729. doi:10.4149/BLL_2022_116
104. Masiuk H, Kopron K, Grumann D, et al. Association of recurrent furunculosis with Panton-Valentine leukocidin and the genetic background of Staphylococcus aureus. J Clin Microbiol. 2010;48(5):1527-1535. doi:10.1128/JCM.02094-09
105. McCaig LF, McDonald LC, Mandal S, Jernigan DB. Staphylococcus aureus-associated skin and soft tissue infections in ambulatory care. Emerging Infect Dis. 2006;12(11): 1715–1723
106. McDonald M, Dougall A, Holt D, et al. Use of a single-nucleotide polymorphism genotyping system to demonstrate the unique epidemiology of methicillin-resistant Staphylococcus aureus in remote aboriginal communities. J Clin Microbiol. 2006;44(10):3720-3727. doi:10.1128/JCM.00836-06
107. Meislin HW, Lerner SA, Graves MH, et al. Cutaneous abscesses: anaerobic and aerobic bacteriology and outpatient management. Ann Intern Med. 1977;87:145-149
108. Mercer DK, Katvars LK, Hewitt F, Smith DW, Robertson J, O'Neil DA. NP108, an antimicrobial polymer with activity against methicillin- and mupirocin-resistant Staphylococcus aureus. Antimicrob Agents Chemother. 2017;61(9):e00502-17. Published 2017 Aug 24. doi:10.1128/AAC.00502-17
109. Mergenhagen K, Starr K, Wattengel B, Lesse A, Sumon Z, Sellick J. Determining the utility of methicillin-resistant Staphylococcus aureus nares screening in antimicrobial stewardship. Clin Infect Dis. 2020;71(5):1142-1148. doi:10.1093/ cid/ciz974.
110. Merritt C, Haran JP, Mintzer J, Stricker J, Merchant RC. All purulence is local— epidemiology and management of skin and soft tissue infections in three urban emergency departments. BMC Emerg Med 2013;13: 26. http://dx.doi.org/10.1186/1471-227X-13-26.
111. Meumann EM, Andersson P, Yeaman F, et al. Whole genome sequencing to investigate a putative outbreak of the virulent community-associated methicillin-resistant Staphylococcus aureus ST93 clone in a remote Indigenous community. Microb Genom. 2016;2(12):e000098. Published 2016 Dec 12. doi:10.1099/mgen.0.000098
112. Miko BA, Herzig CT, Mukherjee DV, et al. Is environmental contamination associated with Staphylococcus aureus clinical infection in maximum security prisons?. Infect Control Hosp Epidemiol. 2013;34(5):540-542. doi:10.1086/670218
113. Millar EV, Chen WJ, Schlett CD, et al. Frequent use of chlorhexidine-based body wash associated with a reduction in methicillin-resistant Staphylococcus aureus nasal colonization among military trainees. Antimicrob Agents Chemother. 2015;59(2):943-949. doi:10.1128/AAC.03993-14
114. Millar EV, Rice GK, Schlett CD, et al. Genomic epidemiology of MRSA infection and colonization isolates among military trainees with skin and soft tissue infection. Infection. 2019;47(5):729-737. doi:10.1007/s15010-019-01282-w
115. Miller LG, Perdreau-Remington F, Bayer AS, et al. Clinical and epidemiologic characteristics cannot distinguish community-associated methicillin-resistant Staphylococcus aureus infection from methicillin-susceptible S. aureus infection: a prospective investigation. Clin Infect Dis. 2007;44(4):471-482. doi:10.1086/511033
116. Miller LG, Eells SJ, Taylor AR, et al. Staphylococcus aureus colonization among household contacts of patients with skin infections: risk factors, strain discordance, and complex ecology. Clin Infect Dis. 2012;54(11):1523-1535. doi:10.1093/cid/cis213
117. Mithoe D, Rijnders MI, Roede BM, Stobberingh E, Möller AV. Prevalence of community-associated meticillin-resistant Staphylococcus aureus and Panton-Valentine leucocidin-positive S. aureus in general practice patients with skin and soft tissue infections in the northern and southern regions of The Netherlands. Eur J Clin Microbiol Infect Dis. 2012;31(3):349-356. doi:10.1007/s10096-011-1316-9
118. Methicillin-resistant Staphylococcus aureus infections in correctional facilities: Georgia, California, and Texas, 2001-2003. MMWR Morb Mortal Wkly Rep. 2003;52:992-996.
119. Moffitt K, Cheung E, Yeung T, Stamoulis C, Malley R. Analysis of Staphylococcus aureus transcriptome in pediatric soft tissue abscesses and comparison to murine infections. Infect Immun. 2021;89(4):e00715-20. Published 2021 Mar 17. doi:10.1128/IAI.00715-20
120. Mokhtari A, Ebrahimi-Kahrizangi A, and Hasani P. Genomic identification of toxic shock syndrome producing and methicillin resistant Staphylococcus aureus strains in human and sheep isolates. Journal of the Hellenic Veterinary Medical Society, 69(2), 941–950.2018. https://doi.org/10.12681/jhvms.18016
121. Monaco M, Pedroni P, Sanchini A, Bonomini A, Indelicato A, Pantosti A. Livestock-associated methicillin-resistant Staphylococcus aureus responsible for human colonization and infection in an area of Italy with high density of pig farming. BMC Infect Dis. 2013;13:258. Published 2013 Jun 3. doi:10.1186/1471-2334-13-258
122. Moran GJ, Krishnadasan A, Gorwitz RJ, et al. Methicillin-resistant S aureus infections among patients in the emergency department. N Engl J Med. 2006;355(7):666 – 674
123. Mork RL, Hogan PG, Muenks CE, et al. Comprehensive modeling reveals proximity, seasonality, and hygiene practices as key determinants of MRSA colonization in exposed households. Pediatr Res. 2018;84(5):668-676. doi:10.1038/s41390-018-0113-x
124. Nagaraju U, Bhat G, Kuruvila M, Pai GS, Jayalakshmi, Babu RP. Methicillin-resistant Staphylococcus aureus in community-acquired pyoderma. Int J Dermatol. 2004;43(6):412-414. doi:10.1111/j.1365-4632.2004.02138.x
125. Naimi T, LeDell KH, Como-Sabetti K, Borchardt SM, Boxrud DJ, Etienne J, et al. Comparison of community- and health care-associated methicillinresistant Staphylococcus aureus infection. JAMA 2003; 290:2976–2984
126. Nakamura MM, Rohling KL, Shashaty M, et al. Prevalence of methicillin-resistant Staphylococcus aureus nasal carriage in the community pediatric population. Pediatr Infect Dis J. 2002;21:917–921
127. Namura S, Nishijima S, Higashida T, Asada Y. Staphylococcus aureus isolated from nostril anteriors and subungual spaces of the hand: comparative study of medical staff, patients, and normal controls. J Dermatol. 1995;22(3):175-180. doi:10.1111/j.1346-8138.1995.tb03366.x
128. Nguyen DM, Mascola L, Brancoft E. Recurring methicillin-resistant Staphylococcus aureus infections in a football team. Emerg Infect Dis. 2005;11(4):526-532. doi:10.3201/eid1104.041094
129. Nilsson P, Ripa T. Staphylococcus aureus throat colonization is more frequent than colonization in the anterior nares. J Clin Microbiol 2006; 44:3334-9.
130. Nishijima S, Kurokawa I. Antimicrobial resistance of Staphylococcus aureus isolated from skin infections. Int J Antimicrob Agents. 2002;19:241-243.
131. Nurjadi D, Fleck R, Lindner A, et al. Import of community-associated, methicillin-resistant Staphylococcus aureus to Europe through skin and soft-tissue infection in intercontinental travellers, 2011-2016. Clin Microbiol Infect. 2019;25(6):739-746. doi:10.1016/j.cmi.2018.09.023
132. Ogura J, Inayama Y, Sasamoto N, et al. Vertical transmission of methicillin-resistant Staphylococcus aureus at delivery and its clinical impact: An observational, prospective cohort study. Acta Obstet Gynecol Scand. 2021;100(11):1986-1994. doi:10.1111/aogs.14240
133. Okoye MT, Sofowora K, Singh A, Fergie J. Decrease in the prevalence of methicillin-resistant Staphylococcus aureus nasal colonization of children admitted to Driscoll Children's Hospital. Pediatr Infect Dis J. 2019;38(2):e34-e36. doi:10.1097/INF.0000000000002116
134. Okuma K, Iwakawa K, Turnidge JD, et al. Dissemination of new methicillin-resistant Staphylococcus aureus clones in the community. J Clin Microbiol. 2002;40:4289-4294.
135. Oliva A, Lichtner M, Mascellino MT, et al. Study of methicillin-resistant Staphylococcus aureus (MRSA) carriage in a population of HIV-negative migrants and HIV-infected patients attending an outpatient clinic in Rome. Ann Ig. 2013;25(2):99-107. doi:10.7416/ai.2013.1911
136. Orellana RC, Hoet AE, Bell C, et al. Methicillin-resistant Staphylococcus aureus in Ohio EMS Providers: A Statewide Cross-sectional Study. Prehosp Emerg Care. 2016;20(2):184-190. doi:10.3109/10903127.2015.1076098
137. Packer S, Pichon B, Thompson S, et al. Clonal expansion of community-associated meticillin-resistant Staphylococcus aureus (MRSA) in people who inject drugs (PWID): prevalence, risk factors and molecular epidemiology, Bristol, United Kingdom, 2012 to 2017. Euro Surveill. 2019;24(13):1800124. doi:10.2807/1560-7917.ES.2019.24.13.1800124
138. Patil R, Baveja S, Nataraj G, et al. Prevalence of methicillin-resistant Staphylococcus aureus (MRSA) in community-acquired primary pyodermaIndian J Dermatol Venereol Leprol. 2006; 72:126-8.
139. Pelzek AJ, Shopsin B, Radke EE, et al. Human memory B cells targeting Staphylococcus aureus exotoxins are prevalent with skin and soft tissue infection. mBio. 2018;9(2):e02125-17. Published 2018 Mar 13. doi:10.1128/mBio.02125-17
140. Petry N, Montgomery A, Hammer K, Lo T. Methicillinresistant Staphylococcus aureus (MRSA) screening upon inpatient hospital admission: is there concordance between nasal swab results and samples taken from skin and soft tissue? Infect Control Hosp Epidemiol. 2020;41(11):1298-1301. doi:10.1017/ice.2020.345.
141. Purcell K, Fergie J. Epidemic of community-acquired methicillin-resistant Staphylococcus aureus infections: a 14-year study at Driscoll Children’s Hospital. Arch Pediatr Adolesc Med. 2005;159:980 –985.
142. Rahimian J, Khan R, LaScalea KA. Does nasal colonization or mupirocin treatment affect recurrence of methicillin-resistant Staphylococcus aureus skin and skin structure infections?. Infect Control Hosp Epidemiol. 2007;28(12):1415-1416. doi:10.1086/523273
143. Rahman MM, Amin KB, Rahman SMM, et al. Investigation of methicillin-resistant Staphylococcus aureus among clinical isolates from humans and animals by culture methods and multiplex PCR. BMC Vet Res. 2018;14(1):300. Published 2018 Oct 3. doi:10.1186/s12917-018-1611-0
144. Ray G, Suaya J, Baxter R. Incidence, microbiology and patient characteristics of skin and soft-tissue infections in a US population: a retrospective population-based study. BMC Infect Dis. 2013;13:252. doi:10.1186/1471-2334-13-252.
145. Read TD, Petit RA 3rd, Yin Z, Montgomery T, McNulty MC, David MZ. USA300 Staphylococcus aureus persists on multiple body sites following an infection. BMC Microbiol. 2018;18(1):206. Published 2018 Dec 5. doi:10.1186/s12866-018-1336-z
146. Renwick L, Hardie A, Girvan EK, et al. Detection of meticillin-resistant Staphylococcus aureus and Panton-Valentine leukocidin directly from clinical samples and the development of a multiplex assay using real-time polymerase chain reaction. Eur J Clin Microbiol Infect Dis. 2008;27(9):791-796. doi:10.1007/s10096-008-0503-9
147. Ridgway JP, Peterson LR, Brown EC, et al. Clinical significance of methicillin-resistant Staphylococcus aureus colonization on hospital admission: one-year infection risk. PLoS One. 2013;8(11):e79716. Published 2013 Nov 20. doi:10.1371/journal.pone.0079716
148. Romano R, Lu D, Holtom P. Outbreak of community-acquired methicillin-resistant Staphylococcus aureus skin infections among a collegiate football team. J Athl Train. 2006;41(2):141-145.
149. Rosenthal A, White D, Churilla S, Brodie S, Katz KC. Optimal surveillance culture sites for detection of methicillin-resistant Staphylococcus aureus in newborns. J Clin Microbiol. 2006;44(11):4234 – 4236
150. Salmanov A, Vozianov S, Kryzhevsky V, Litus O, Drozdova A, Vlasenko I. Prevalence of healthcare-associated infections and antimicrobial resistance in acute care hospitals in Kyiv, Ukraine. J Hosp Infect. 2019;102(4):431-437. doi:10.1016/j.jhin.2019.03.008
151. Ridgway JP, Peterson LR, Brown EC, et al. Clinical significance of methicillin-resistant Staphylococcus aureus colonization on hospital admission: one-year infection risk. PLoS One. 2013;8(11):e79716. Published 2013 Nov 20. doi:10.1371/journal.pone.0079716
152. Sattler CA, Mason EO Jr, Kaplan SL. Prospective comparison of risk factors and demographic and clinical characteristics of community-acquired, methicillin-resistant versus methicillin-susceptible Staphylococcus aureus infection in children. Pediatr Infect Dis J. 2002;21:910-917.
153. Sdougkos G, Chini V, Papanastasiou DA, et al. Community-associated Staphylococcus aureus infections and nasal carriage among children: molecular microbial data and clinical characteristics. Clin Microbiol Infect. 2008;14(11):995-1001. doi:10.1111/j.1469-0691.2008.02064.x
154. Shaban RZ, Li C, O'Sullivan MVN, et al. Outbreak of community-acquired Staphylococcus aureus skin infections in an Australian professional football team. J Sci Med Sport. 2021;24(6):520-525. doi:10.1016/j.jsams.2020.11.006
155. Sherertz RJ, Reagan DR, Hampton KD, et al. A cloud adult: the Staphylococcus aureus-virus interaction revisited. Ann Intern Med. 1996;124(6):539-547. doi:10.7326/0003-4819-124-6-199603150-00001
156. Shet A, Mathema B, Mediavilla JR, et al. Colonization and subsequent skin and soft tissue infection due to methicillin-resistant Staphylococcus aureus in a cohort of otherwise healthy adults infected with HIV type 1. J Infect Dis. 2009;200(1):88-93. doi:10.1086/599315
157. Shukla SK, Karow ME, Brady JM, et al. Virulence genes and genotypic associations in nasal carriage, community-associated methicillin-susceptible and methicillin-resistant USA400 Staphylococcus aureus isolates. J Clin Microbiol. 2010;48(10):3582-3592. doi:10.1128/JCM.00657-10
158. Sligl W, Taylor G, Gibney RN, Rennie R, Chiu L. Methicillin-resistant Staphylococcus aureus in a Canadian intensive care unit: delays in initiating effective therapy due to the low prevalence of infection. Can J Infect Dis Med Microbiol 2007; 18:139–143
159. Smith TC, Hellwig EJ, Wardyn SE, Kates AE, Thapaliya D. Longitudinal case series of Staphylococcus aureus colonization and infection in two cohorts of rural Iowans. Microb Drug Resist. 2018;24(4):455-460. doi:10.1089/mdr.2017.0124
160. Sobhy N, Aly F, Abd El Kader O, Ghazal A, Elbaradei A. Community-acquired methicillin-resistant Staphylococcus aureus from skin and soft tissue infections (in a sample of Egyptian population): analysis of mec gene and staphylococcal cassette chromosome. Braz J Infect Dis. 2012;16(5):426-431. doi:10.1016/j.bjid.2012.08.004
161. Stenstrom R, Grafstein E, Romney M, et al. Prevalence of and risk factors for methicillin-resistant Staphylococcus aureus skin and soft tissue infection in a Canadian emergency department. CJEM 2009; 11:430–438
162. Stevens AM, Hennessy T, Baggett HC, Bruden D, Parks D, Klejka J. Methicillin-Resistant Staphylococcus aureus carriage and risk factors for skin infections, Southwestern Alaska, USA. Emerg Infect Dis. 2010;16(5):797-803. doi:10.3201/eid1605.091851
163. Suh K, Toye B, Jessamine P, Chan F, Ramotar K. Epidemiology of methicillinresistant Staphylococcus aureus in three Canadian tertiary-care centers. Infect Control Hosp Epidemiol 1998; 19:395–400.
164. Summanen PH, Talan DA, Strong C, et al. Bacteriology of skin and soft-tissue infections: comparison of infections in intravenous drug users and individuals with no history of intravenous drug use. Clin Infect Dis. 1995;20(Suppl 2):S279-282.
165. Szumowski JD, Wener KM, Gold HS, et al. Methicillin-resistant Staphylococcus aureus colonization, behavioral risk factors, and skin and soft-tissue infection at an ambulatory clinic serving a large population of HIV-infected men who have sex with men. Clin Infect Dis. 2009;49(1):118-121. doi:10.1086/599608
166. Taguchi H, Noguchi N, Nakaminami H, Sasatsu M. Panton-Valentine leucocidin-positive MRSA infection in inpatients at a tertiary care centre in Tokyo, Japan. J Hosp Infect. 2010;76(3):268-269. doi:10.1016/j.jhin.2010.04.013
167. Talan DA, Krishnadasan A, Gorwitz RJ, et al. Comparison of Staphylococcus aureus from skin and soft-tissue infections in US emergency department patients, 2004 and 2008. Clin Infect Dis 2011;53:144 149. http://dx.doi.org/10.1093/cid/cir308.
168. Tang CT, Nguyen DT, Ngo TH, et al. An outbreak of severe infections with community-acquired MRSA carrying the Panton-Valentine leukocidin following vaccination. PLoS One. 2007;2(9):e822. Published 2007 Sep 5. doi:10.1371/journal.pone.0000822
169. Tang CS, Wang CC, Huang CF, Chen SJ, Tseng MH, Lo WT. Antimicrobial susceptibility of Staphylococcus aureus in children with atopic dermatitis. Pediatr Int. 2011;53(3):363-367. doi:10.1111/j.1442-200X.2010.03227.x
170. Tenover FC, McAllister S, Fosheim G, et al. Characterization of Staphylococcus aureus isolates from nasal cultures collected from individuals in the United States in 2001 to 2004. J Clin Microbiol 2008;46:2837–2841. http://dx.doi.org/10.1128/JCM.00480-08.
171. Terpenning MS, Bradley SF, Wan JY, Chenoweth CE, Jorgensen KA, Kauffman CA. Colonization and infection with antibiotic-resistant bacteria in a long-term care facility. J Am Geriatr Soc. 1994;42(10):1062-1069. doi:10.1111/j.1532-5415.1994.tb06210.x
172. Thind P, Prakash SK, Wadhwa A, et al. Bacteriological profile of communityacquired pyodermas with special reference to methicillin resistant Staphylococcus aureus. Indian J Dermatol Venereol Leprol. 2010; 76: 572-4.
173. Tinelli M, Monaco M, Vimercati M, Ceraminiello A, Pantosti A. Methicillin-susceptible Staphylococcus aureus in skin and soft tissue infections, Northern Italy. Emerg Infect Dis. 2009;15(2):250-257. doi:10.3201/eid1502.080010
174. Tobin JN, Hower S, D'Orazio BM, et al. Comparative effectiveness study of home-based interventions to prevent CA-MRSA infection recurrence. Antibiotics (Basel). 2021;10(9):1105. Published 2021 Sep 13. doi:10.3390/antibiotics10091105
175. Tomasz A, Nachman S, Leaf H.. Stable classes of phenotypic expression in methicillin-resistant clinical isolates of staphylococci. Antimicrob Agents Chemother 1991;35:124 –129. http://dx.doi.org/10.1128/AAC.35.1 .124.
176. Trościańczyk A, Nowakiewicz A, Kasela M, et al. Multi-Host pathogen Staphylococcus aureus-Epidemiology, drug resistance and occurrence in humans and animals in Poland. Antibiotics (Basel). 2023;12(7):1137. Published 2023 Jun 30. doi:10.3390/antibiotics12071137
177. Venniyil PV, Ganguly S, Kuruvila S, et al. A study of community-associated methicillin-resistant Staphylococcus aureus in patients with pyoderma. Indian Dermatol Online J. 2016; 7:159-63.
178. Wagenlehner FM, Naber KG, Bambl E, et al. Management of a large healthcare-associated outbreak of Panton-Valentine leucocidin-positive meticillin-resistant Staphylococcus aureus in Germany. J Hosp Infect. 2007;67(2):114-120. doi:10.1016/j.jhin.2007.07.006
179. Walraven CJ, Lingenfelter E, Rollo J, Madsen T, Alexander DP. Diagnostic and therapeutic evaluation of community-acquired methicillin-resistant Staphylococcus aureus (MRSA) skin and soft tissue infections in the emergency department. J Emerg Med 2012; 42: 392-9.
180. Tang CS, Wang CC, Huang CF, Chen SJ, Tseng MH, Lo WT. Antimicrobial susceptibility of Staphylococcus aureus in children with atopic dermatitis. Pediatr Int. 2011;53(3):363-367. doi:10.1111/j.1442-200X.2010.03227.x
181. Weintrob A, Bebu I, Agan B, et al. Randomized, double-blind, placebo-controlled study on decolonization procedures for methicillin-resistant Staphylococcus aureus (MRSA) among HIV-infected adults. PLoS One. 2015;10(5):e0128071. Published 2015 May 27. doi:10.1371/journal.pone.0128071
182. Wenzel RP, Bearman G, Edmond MB. Screening for MRSA: a flawed hospital infection control intervention. Infect Control Hosp Epidemiol. 2008;29(11):1012–1018
183. Whitman TJ, Herlihy RK, Schlett CD, et al. Chlorhexidine-impregnated cloths to prevent skin and soft-tissue infection in Marine recruits: a cluster-randomized, double-blind, controlled effectiveness trial. Infect Control Hosp Epidemiol. 2010;31(12):1207-1215. doi:10.1086/657136
184. Whitman TJ, Schlett CD, Grandits GA, et al. Chlorhexidine gluconate reduces transmission of methicillin-resistant Staphylococcus aureus USA300 among Marine recruits. Infect Control Hosp Epidemiol. 2012;33(8):809-816. doi:10.1086/666631
185. Wibbenmeyer LA, Kealey GP, Latenser BA, et al. Emergence of the USA300 strain of methicillin-resistant Staphylococcus aureus in a burn-trauma unit. J Burn Care Res. 2008;29(5):790-797. doi:10.1097/BCR.0b013e3181848b8f
186. Williams DJ, Cooper WO, Kaltenbach LA, et al. Comparative effectiveness of antibiotic treatment strategies for pediatric skin and soft-tissue infections. Pediatrics. 2011;128(3):e479-e487. doi:10.1542/peds.2010-3681
187. Yang ES, Tan J, Eells S, Rieg G, Tagudar G, Miller LG. Body site colonization in patients with community-associated methicillin-resistant Staphylococcus aureus and other types of S. aureus skin infections. Clin Microbiol Infect. 2010;16(5):425-431. doi:10.1111/j.1469-0691.2009.02836.x
188. Zafar U, Johnson LB, Hanna M, et al. Prevalence of nasal colonization among patients with community-associated methicillin resistant Staphylococcus aureus infection and their household contacts. Infect Control Hosp Epidemiol. 2007;28:966 –969.
189. Zanger P, Nurjadi D, Schleucher R, Scherbaum H, Wolz C, Kemsner PG, et al. Import and spread of Panton–Valentine leukocidinpositive Staphylococcus aureus through nasal carriage and skin infections in travelers returning from the tropics and subtropics. Clin Infect Dis 2012;54:483 – 92.
190. Zhou W, Jin Y, Teng G, et al. Comparative analysis of genomic characteristics, virulence and fitness of community-associated Staphylococcus aureus ST121 clone causing fatal diseases in China and other CA-MRSA clones. Virulence. 2023;14(1):2242547. doi:10.1080/21505594.2023.2242547
